# Mitochondrial function in skeletal myofibers is controlled by a TRF2‐SIRT3 axis over lifetime

**DOI:** 10.1111/acel.13097

**Published:** 2020-01-28

**Authors:** Jérôme D. Robin, Maria‐Sol Jacome Burbano, Han Peng, Olivier Croce, Jean Luc Thomas, Camille Laberthonniere, Valerie Renault, Liudmyla Lototska, Mélanie Pousse, Florent Tessier, Serge Bauwens, Waiian Leong, Sabrina Sacconi, Laurent Schaeffer, Frédérique Magdinier, Jing Ye, Eric Gilson

**Affiliations:** ^1^ Université Côte d'Azur CNRS Inserm Institut for Research on Cancer and Aging, Nice (IRCAN) Medical School of Nice Nice France; ^2^ Marseille Medical Genetics (MMG) U1251 Aix Marseille University Marseille France; ^3^ International Research Laboratory in “Hematology, Cancer and Aging” Shanghai Jiao Tong University School of Medicine/Ruijin Hospital/CNRS/Inserm/Nice University Pôle Sino‐Français de Recherche en Sciences du Vivant et Génomique Shanghai Ruijin Hospital Shanghai China; ^4^ Neuromuscular Differentiation Group Institut NeuroMyoGene (INMG) UMR5310 Inserm U1217 Ecole Normale Supérieure de Lyon Lyon France; ^5^ Peripheral Nervous System, Muscle and ALS Neuromuscular & ALS Center of Reference FHU Oncoage Pasteur 2 Nice University Hospital Nice France; ^6^ Department of Medical Genetics Archet 2 Hospital FHU Oncoage CHU of Nice Nice France

**Keywords:** aging, mitochondria, postmitotic cells, skeletal muscle, telomeres

## Abstract

Telomere shortening follows a developmentally regulated process that leads to replicative senescence of dividing cells. However, whether telomere changes are involved in postmitotic cell function and aging remains elusive. In this study, we discovered that the level of the TRF2 protein, a key telomere‐capping protein, declines in human skeletal muscle over lifetime. In cultured human myotubes, TRF2 downregulation did not trigger telomere dysfunction, but suppressed expression of the mitochondrial Sirtuin 3 gene (*SIRT3*) leading to mitochondrial respiration dysfunction and increased levels of reactive oxygen species. Importantly, restoring the Sirt3 level in TRF2‐compromised myotubes fully rescued mitochondrial functions. Finally, targeted ablation of the *Terf2* gene in mouse skeletal muscle leads to mitochondrial dysfunction and sirt3 downregulation similarly to those of TRF2‐compromised human myotubes. Altogether, these results reveal a TRF2‐SIRT3 axis controlling muscle mitochondrial function. We propose that this axis connects developmentally regulated telomere changes to muscle redox metabolism.

## Graphical abstract

The level of the TRF2 protein, a key telomere‐capping protein, declines in human skeletal muscle over lifetime. In cultured human myotubes and in mouse skeletal muscle, TRF2 downregulation did not trigger telomere dysfunction, but suppressed expression of the mitochondrial Sirtuin 3 gene (SIRT3) leading to mitochondrial respiration dysfunction and increased levels of reactive oxygen species. Altogether, our results reveal a TRF2‐SIRT3 axis controling muscle mitochondrial function.

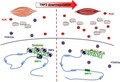

## 1 INTRODUCTION

Aging is characterized by an overall decline in the maintenance of tissue homeostasis, organ function, and stress response. In tissues, the accumulation of senescent cells (i.e., permanent cell cycle arrest in response to various types of stress or tissue remodeling) has emerged as an important contributor to aging, mainly through nonautonomous cell mechanisms driving chronic inflammation and tissue degeneration (Baker et al., 2016; van Deursen, 2014; López‐Otín, Blasco, Partridge, Serrano, & Kroemer, 2013). Tissue aging is also characterized by the progressive decline in the function of long‐lived postmitotic cells, such as myofibers and neurons.

Normal skeletal muscle aging is mainly driven by changes occurring in postmitotic, fully differentiated multinucleated myofibers such as progressive alterations in transcriptional and metabolic programs, dismantlement of neuromuscular junctions, and progressive loss of the most powerful fast fibers (Bua et al., 2006; Lexell, 1995; Miljkovic, Lim, Miljkovic, & Frontera, 2015; Renault et al., 2002). A decline of muscle stem cells was associated with the etiology of sarcopenia, an age‐associated loss of skeletal muscle mass and strength (Snijders & Parise, 2017). However, sarcopenia cannot be only explained by the loss of stem cell over lifetime (Fry et al., 2015). Thus, the age‐dependent mechanisms triggering muscle aging and their links to the cellular senescence pathways remain largely unknown (Fry et al., 2015; Lexell, 1995; Miljkovic et al., 2015; Renault et al., 2002).

Unlike stem cells, wherein division and mobilization can be considered as a temporary rejuvenation mechanism due to the accumulation of molecular alterations such as telomere shortening, long‐lived postmitotic cells mainly rely on their capacity for repair, quality control, and intracellular renewal (i.e., autophagy, mitophagy, and proteostasis). It is generally accepted that the age‐dependent increase in reactive oxygen species (ROS) production by dysfunctional mitochondria and the inherent inability of autophagy and other cellular mechanisms to remove damaged molecules are responsible for progressive functional decline in long‐lived postmitotic cells (Bua et al., 2006; Linnane, Marzuki, Ozawa, & Tanaka, 1989; Rubinsztein, Mariño, & Kroemer, 2011). Therefore, the current model for aging in these cells relies on the accumulation of molecular damage, mitochondrial dysfunctions, and increased ROS production, which progressively alter their transcriptional program, metabolism, and functional performance. Whether these aging events result from developmentally regulated mechanisms or a simple cumulative effect is still elusive.

Among known mechanisms of aging, the unrelenting erosion of telomeres acts as a developmentally regulated process driving replicative senescence (Gilson & Géli, 2007; López‐Otín et al., 2013). Besides requiring the telomerase to be fully replicated, telomeres have several peculiarities and adopt specific chromatin conformations involving telomeric DNA looping (t‐loop), the binding of specific protective factors such as the shelterin complex and noncoding RNA (i.e., TERRA; Gilson & Géli, 2007). In humans, the shelterin complex comprises six proteins: the telomeric repeat‐binding factor 1 (TRF1; also known as TERF1); the telomeric repeat‐binding factor 2 (TRF2; also known as TERF2); the protection of telomeres 1 (POT1); the ACD shelterin complex subunit and telomerase recruitment factor (TPP1); the repressor activator protein 1 (RAP1); and the TRF1‐TRF2 interacting nuclear protein 2 (TIN2). This complex binds to telomeres, favors t‐loop formation, and prevents unwanted activation of the DNA damage response (DDR).

The current view on telomere dynamics in long‐lived postmitotic cell aging has been revisited in recent studies that reported telomere shortening in cell differentiation (Flores et al., 2008), muscle and fat aging (Carneiro et al., 2016; Daniali et al., 2013), stressed neurons (Zglinicki, 2002), and pathologic myocardium (Chang et al., 2016). Consistent with the existence of a telomere maintenance mechanism specific to postmitotic cells, yeast telomeres undergo a profound nuclear reorganization when cells enter into quiescence (Guidi et al., 2015), a process reminiscent of the postmitotic state in multicellular species.

Notably, a wealth of data indicates that two key aging hallmarks (López‐Otín et al., 2013), telomere and oxidative metabolism, are intimately connected. Due to its high content in guanine, telomeric DNA is easily oxidized, leading to the accumulation of 8‐oxo‐guanine, which disrupts the binding of telomere protective factors (Opresko, Fan, Danzy, Wilson, & Bohr, 2005) and prevents extension by telomerase (Aeby, Ahmed, Redon, Simanis, & Lingner, 2016; Fouquerel et al., 2016). Concomitantly, telomeres shorten under oxidative stress, whereas antioxidant treatments are associated with their reduced erosion (Ahmed & Lingner, 2018; Passos et al., 2007; Zglinicki, 2002), and antioxidant proteins are specifically associated with telomeres (Aeby et al., 2016). The catalytic subunit of telomerase, TERT, can be localized in the mitochondria where it plays a protective role for metabolism and DNA maintenance (Ahmed et al., 2008). Telomerase RNA can be processed within the mitochondria (Cheng et al., 2018), and some shelterin subunits have been shown to have mitochondrial functions (Chen et al., 2012; Kim et al., 2017). Telomere dysfunction activates p53, which in turn binds and represses PGC1‐*α* and PGC1‐*β* promoters leading to mitochondrial dysfunction (Chang et al., 2016; Sahin et al., 2011). Overall, it appears that telomere dysfunction triggers mitochondrial alteration and *vice versa*. It is a tempting hypothesis that such an “explosive” regulatory loop might drive long‐lived postmitotic cell aging. In further support for a role of telomeres in postmitotic cell aging are the differential expression of the TRF2 shelterin subunit in cardiomyocytes upon physical exercise or heart failure (Oh et al., 2003)^,^ and the extratelomeric roles of this protein in neuronal gene expression (Robin et al., 2014; Yang et al., 2011; Ye, Renault, Jamet, & Gilson, 2014).

In this study, we explored changes in the telomere state in vivo during muscle development and aging and their functional significance in vitro using differentiated skeletal muscle fibers (myotubes). We found that TRF2 expression and telomeric DNA length start decreasing in human skeletal muscles of young adults, indicating important developmentally regulated changes in telomere composition at the end of muscle development, which are accentuated in elders. Remarkably, *TERF2* downregulation in human myotubes led not to telomere shortening and deprotection but to changes in mitochondrial respiration and elevated ROS levels through a direct mechanism involving transcriptional regulation of the *SIRT3* subtelomeric gene encoding a mitochondrial Sirtuin. Besides, using a transgenic mouse model abolishing *Terf2* expression in skeletal muscle, we did not observe telomeric deprotection but changes in mitochondrial respiration, increased oxidative environment, and Sirt3 downregulation.

## 2 RESULTS

### 2.1 TRF2 level negatively correlates with age in human skeletal muscle biopsies

Rapid muscle cell proliferation occurs during embryogenesis, decreases progressively during the fetal stage, and becomes negligible in early adolescence (Lepper, Conway, & Fan, 2009; Miljkovic et al., 2015; Murphy, Lawson, Mathew, Hutcheson, & Kardon, 2011; Relaix & Zammit, 2012). As a consequence, the proportion of satellite cells decreases from ~30% to 5%–6% between the postnatal period and early teens (Dayanidhi & Lieber, 2014; Romero, Mezmezian, & Fidziańska, 2013; Schultz, 1974). Adult skeletal muscle is mainly composed of postmitotic differentiated multinucleated myofibers and 5%–6% of quiescent satellite cells (Moss & Leblond, 1971; Schultz, Gibson, & Champion, 1978) suggesting a minimum influence of the pool of satellite cells in the composition of skeletal muscle tissue when addressing biological parameters such as telomeres (e.g., length, shelterin complex).

**Figure 1:**
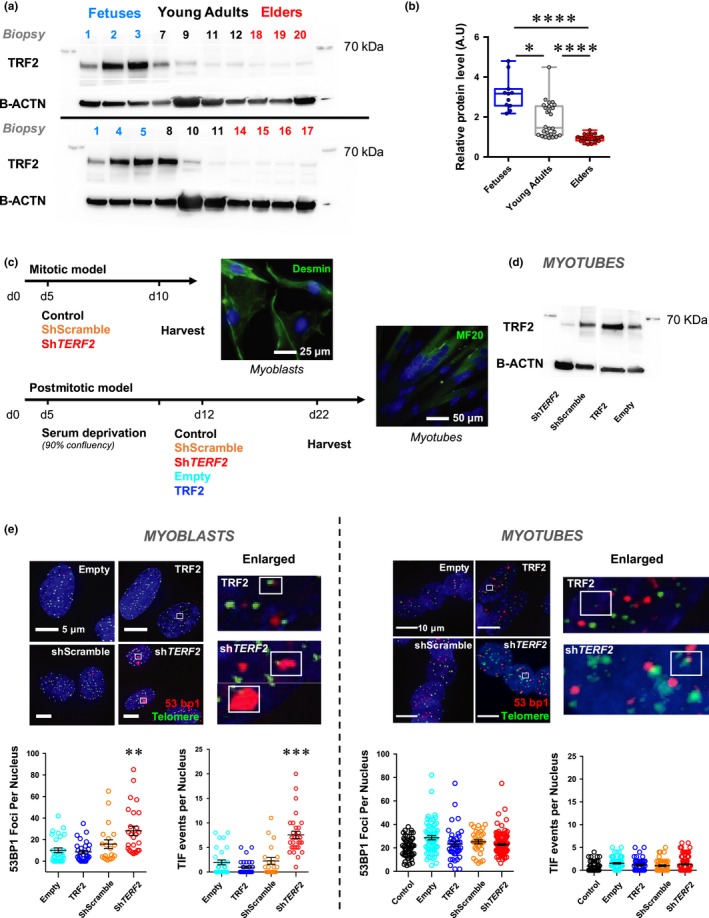
Specific TRF2 downregulation in skeletal muscle and myotubes does not trigger telomere damages. (a) Immunoblots and associated quantifications (in independent duplicates, (b) of whole protein extracts from human biopsies (Figure S1) collected at different ages with antibodies against TRF2 (upper panel) and B‐ACTIN used as a loading control (lower panel). Biopsies were grouped in three categories: fetuses; young adults (17–35 yo); and elders (>60 yo). TRF2 level decreases with age, young adults versus elders, *p* < .001; fetuses versus elders, *p* < .001 (Kruskal–Wallis multiple comparisons test; *α* = 0.05). (c) Schematic representation of the strategy used. Myoblasts stained with anti‐desmin antibody and myotubes with MF20 antibody are shown as illustrations; control refers as the untransduced condition. (d) TRF2 immunoblots of transduced human myotubes using B‐ACTIN as a loading control. € 53BP1 staining and TIFs in transduced myoblasts and myotubes performed using a telomeric probe (PNA, green) and 53BP1 (red) antibody indicating dsDNA damage. Means ± *SEM* are shown. Only foci within multinucleated cells, that is, corresponding to postmitotic myotubes were counted (*n* > 40 nuclei per condition). No statistical difference in myotubes is seen between the different conditions (*TERF2* overexpression or knockdown and respective controls; ANOVA, Kruskal–Wallis multiple comparisons test, α = 0.05). *p < .05; **p < .01; ****p < .001

Using human skeletal muscle biopsies isolated from individuals at different ages (*n* = 20, fetuses to 72‐year‐old adults; Figure S1a), we first confirmed telomere shortening with age as reported previously (Figure S1b; Daniali et al., 2013). In a striking parallel with telomere shortening, we found that TRF2 protein levels decreased markedly during the third decade of life and were negatively correlated with age (Figures 1a‐b and S1c; *R* ^2^ = .6967). Importantly, among the six known shelterin proteins (e.g., TRF1, TRF2, POT1, RAP1, TPP1, and TIN2), only TRF2 levels were significantly associated with this age‐associated pattern (Figure S1d), suggesting a specific role of TRF2 in muscle cell function.

### 2.2 TRF2 downregulation in human myotubes did not trigger telomere damage

To investigate the consequences of the TRF2 downregulation observed in skeletal muscle in early adulthood (Figure 1a–b), we knocked down *TERF2* expression using shRNAs in cultured myotubes. At confluence, primary myoblasts obtained from a human biopsy (Robin, Wright, et al., 2015) were switched to differentiation medium (day 5; 2% horse serum). They formed myotubes at day 10, were transduced with lentiviral particles at day 12, and collected at day 22 (Figure 1c). Concomitantly, transduced subconfluent myoblasts were collected 5 days after transduction. Decreased *TERF2* expression in myotubes to levels similar to those observed in early adult muscles (Figure 1a,d) revealed no major differences in cell myogenesis, fiber morphology, or number of nuclei per fiber, indicating the absence of apoptosis triggered by this downregulation (Figures 1e and S2a–b). Moreover, *TERF2* downregulation is not associated with a significant modulation of other shelterin proteins (Figure S2c). Staining of telomeric induced foci (TIFs; Figure 1e) showed an increased rate of recruitment of DDR factors to telomeres, indicating that *TERF2* downregulation triggered telomere uncapping in proliferative myoblasts, as observed in mitotic cells. Intriguingly, under all conditions tested (i.e., four different shRNAs), *TERF2* depletion did not trigger TIF formation in differentiated myotubes (Figure 1e; *TERF2* shRNA TRCN0000004812; Figure S3a). Consistent with the absence of DDR activation in these differentiated cells, we did not detect phosphorylation of ATM and p53 (Figure S3b), changes in telomere length, or signs of telomere fusion (Figure S3c). Noteworthy, if myotubes do not exhibit an increased rate of TIFs upon *TERF2* downregulation, they have a higher level of basal DNA damages compared to myoblasts (Figure 1e), which likely reflects defects in the DNA repair machinery as previously reported (Vahidi Ferdousi et al., 2014), associated with their postmitotic nature.

### 2.3 TRF2 downregulation in human myotubes induced metabolic changes

**Figure 2:**
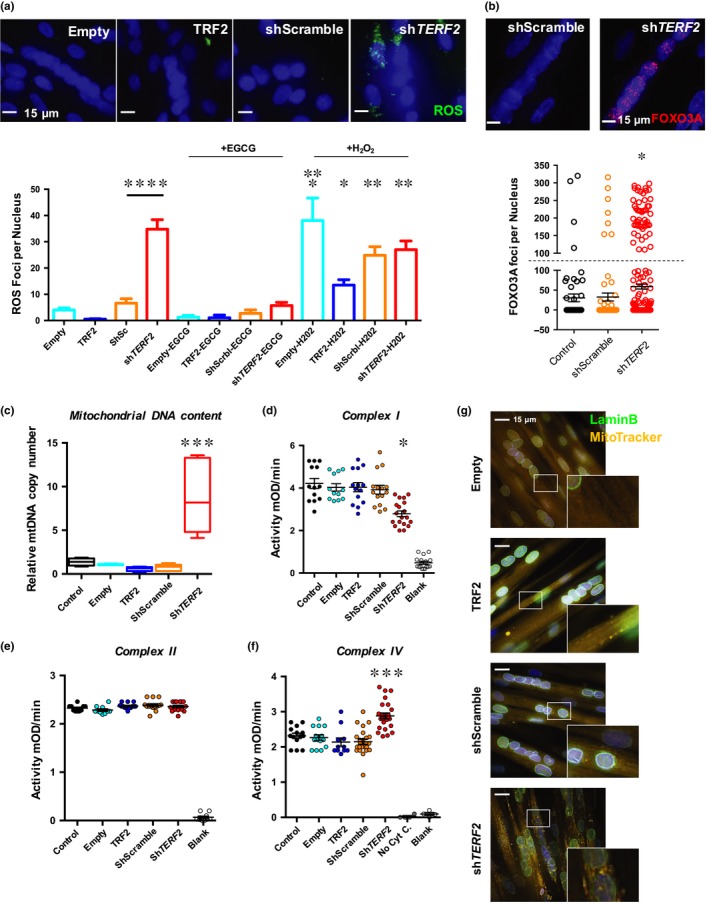
TRF2 knockdown is associated with increased ROS and modifies mitochondrial activity in postmitotic tissues. (a) Detection of ROS foci in transduced myotubes. We report the total number of ROS foci normalized to the number of nuclei. On average, the number of nuclei per cell and DAPI intensities was identical between conditions (Figure S3). *N* > 300 nuclei per condition, means ± *SEM* are shown. Hydrogen peroxide treatment increases ROS foci number (Empty vs. Empty + H_2_O_2_, *p* = .0274; Empty vs. TRF2 + H_2_O_2_, *p* = .0291; Holm–Sidak's multiple comparisons test; α = 0.05). Downregulation of *TERF2* significantly increases ROS (shScramble vs. sh*TERF2*, TRCN0000004812, *p* < .0001; Holm–Sidak's multiple comparisons test; α = 0.05). (b) FOXO3A staining and quantification of foci per nucleus; single nucleus cells were excluded from the analysis. Positive cells correspond to cells exhibiting >100 FOXO3A foci per nucleus (cutoff symbolized by dashed line). *N* = 300 nuclei per condition, means ± *SEM* are shown. *TERF2* knockdown is associated with an increase of positive FOXO3A cells (*n* = 7 vs. *n* = 80 positive nuclei, shScramble vs. sh*TERF2*, respectively, *p* < .0001; chi‐square test; α = 0.05). (c) Relative quantification of mitochondrial DNA content in transduced myotubes. Sh*TERF2*‐transduced myotubes show an increase in mitochondrial DNA content (shScramble vs. sh*TERF2*, *p* = .0003; Kruskal–Wallis multiple comparisons test; α = 0.05). *N* = 6 per condition (biological triplicate in technical duplicate), means ± *SEM* are shown. (d–f) Mitochondrial complex I (d), II (e), and IV (f) activity in transduced myotubes. Sh*TERF2*‐transduced myotubes exhibit specific mitochondrial defects. We report a decreased complex I activity (*p* < .001) and an increased complex IV activity (*p* < .001, Kruskal–Wallis multiple comparison test; α = 0.05). (g) Mitochondrial network in transduced myotubes was analyzed using MitoTracker^®^. An average of 100 z‐stacks was taken for each condition (DeltaVision Elite^®^, GE). Pictures from 10 to 15 independent and randomly chosen microscope fields were taken, treated postacquisition, and deconvoluted with IMARIS. Single nucleus cells were excluded from the analysis. Nuclei were stained using an anti‐lamin B antibody (green) and counterstained with DAPI. A punctate mitochondrial staining is observed in myotubes transduced with sh*TERF2* suggesting mitochondrial fission. Similarly, H_2_O_2_ treatments induce a punctuated mitochondrial staining (Figure S3). *p < .05; **p < .01; ***p < .005, ****p < .001

We then investigated whether *TERF2* inhibition affects other hallmarks of aging, particularly oxidative metabolism (López‐Otín et al., 2013). Upon *TERF2* depletion, we observed a significant increase in ROS levels in myotubes (shScramble vs. sh*TERF2*,* p* < .0001), similar to those induced by H_2_O_2_ treatments (Figures 2a and S4a), as well as increased expression and activity of the *FOXO3A* key oxidative stress response and longevity regulator (Sandri et al., 2004; Figures 2b and S4b–c).

We next examined mitochondria, an important source of ROS. We observed higher levels of mitochondrial DNA (eightfold increase; *p* = .0003; Figure 2c), decreased complex I, and increased complex IV mitochondrial activity (assessing NADH oxidation and cytochrome C reduction, respectively; Figures 2d–f and S4d) associated with a punctuated appearance of the mitochondrial network, suggesting mitochondrial fission (Figure 2g). Importantly, antioxidant treatment decreased ROS production and restored the mitochondrial network (epigallocatechin gallate; Figures 2a and S5a). Overall, these results indicate that TRF2 depletion triggers important mitochondrial changes. The increase in DNA content and the increased activity of complex IV could reflect compensatory mechanisms to complex I deficiency to preserve mitochondrial ATP production, as suggested by others (Kotiadis, Duchen, & Osellame, 2014; Van Bergen et al., 2011). In this context, the interplay between mitochondrial complexes seems independent of complex II, as no statistical differences were found between conditions (Figure 2e).

Given the observed mitochondrial DNA enrichment and modulated activity of the different respiratory complexes (I and IV), we further examined the global oxidative capacity of transduced myotubes by NADH‐tetrazolium reductase (NADH‐TR), a staining used in muscle histology for simple fiber‐type characterization. We found stronger NADH‐TR staining in downregulated *TERF2* myotubes (Figure S5b), suggesting a switch from glycolytic (fast, type II) toward oxidative (slow, type I) fibers, the latter being associated with development, aging, and stronger resistance to oxidative stress (Lexell, 1995; Miljkovic et al., 2015).

### 2.4 TRF2 controls mitochondrial function by activating SIRT3 expression

**Figure 3:**
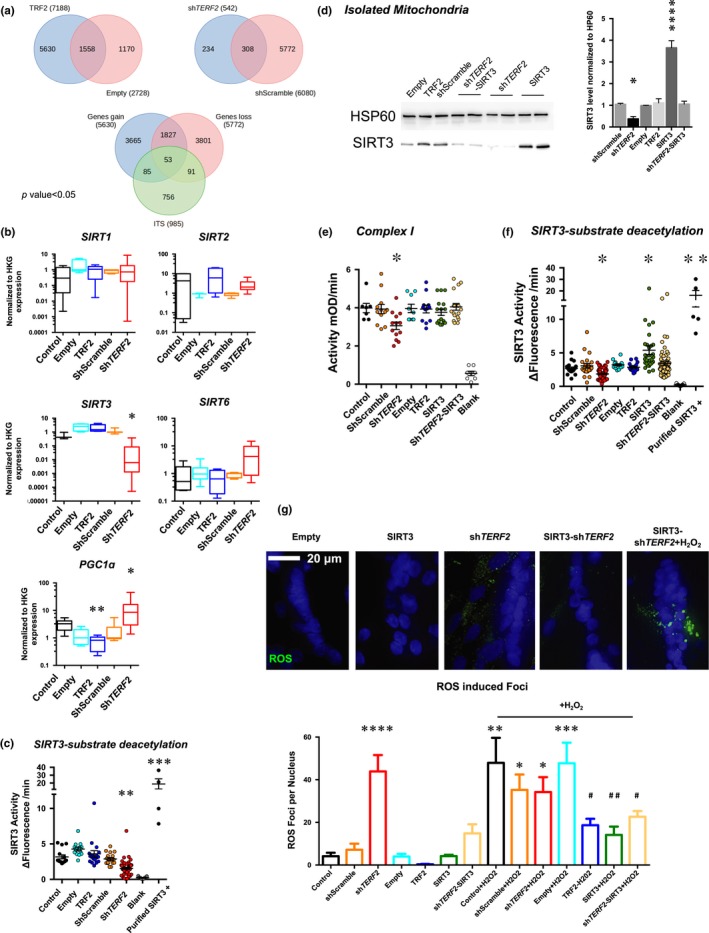
TRF2 binds and modifies expression of the subtelomeric *SIRT3* gene. (a) Venn diagrams generated from a TRF2 ChIP‐Seq performed in myotubes, and all data are uploaded into the GEO database under the accession number http://www.ncbi.nlm.nih.gov/geo/query/acc.cgi?acc=GSE88983. Significant peaks (*p* < .05) were identified and annotated. A differential analysis identified the significant peak that was “lost” or “gained” by TRF2 modulations. A final Venn diagram was then produced using the genes associated with the peaks found only in the shScramble condition (e.g., lost gene); the genes related to the peaks found only in the TRF2 condition (e.g., gained gene) and genes related to ITS by an in silico analysis (see Section 4 for details). The list of 53 genes localized within 100 kb of modulated ChIP peaks and ITS can be found in Table S1. This analysis led to identification of *SIRT3* as a candidate gene. (b) Gene expression quantified by RT‐qPCR in transduced myotubes normalized to three housekeeping genes (HKG: *HPRT*, *PPIA*, and *GAPDH*; ΔΔCt method). *N* = 6 per condition (technical duplicates of biological triplicates), means ± *SEM* with associated statistical significance are reported (Kruskal–Wallis multiple comparisons test; α = 0.05). *TERF2* depletion reduces transcription of *SIRT3* without modulating other main Sirtuins, correlates with *PGC1α* upregulation, and is associated with reduced SIRT3 activity as reported in (c) SIRT3 activity in enriched mitochondria extracts from transduced myotubes. A purified SIRT3 recombinant protein was used as positive control (Kruskal–Wallis multiple comparisons test; α = 0.05). (d–g) *SIRT3* rescue experiments and immunoblots of enriched mitochondria extracts from transduced human myotubes using HSP60 as a loading control (d). *TERF2‐*modulated expression results in decreased SIRT3 level (shScramble vs. sh*TERF2*, *p* = .0425). (e–f) Mitochondrial complex I (e) and SIRT3 activity (f) in mitochondrial extract from transduced myotubes. *SIRT3* overexpression in TRF2‐depleted myotubes restores the mitochondrial‐associated activity (shScramble vs. sh*TERF2*‐SIRT3, *p* > .9; in both assays, Holm–Sidak's multiple comparisons test; α = 0.05, Figure S5). (g) ROS foci in transduced myotubes. We report the total number of ROS foci normalized to the number of nuclei (Figure S6). *N* > 400 nuclei per condition, means ± *SEM* are shown. *SIRT3* overexpression decreases ROS foci number upon *TERF2* downregulation (shScramble vs. sh*TERF2*‐SIRT3, *p* = .496; shScramble vs. sh*TERF2*, <.0001; Holm–Sidak's multiple comparisons test; α = 0.05) and protects myotubes under H_2_O_2_ treatment (Empty + H_2_O_2_ vs. SIRT3 + H_2_O_2_, *p* = .0034; ShScramble + H_2_O_2_ vs. SIRT3‐sh*TERF2*, *p* = .039; Holm–Sidak's multiple comparisons test; α = 0.05). *p < .05; **p < .01; ***p < .001; *****p < .0001; ^#^ *p* < .05; ^##^ *p* < .01 (H_2_O_2_ conditions)

Considering that the role of TRF2 in mitochondrial function appears to be independent of telomere protection and that TRF2 occupies extra telomeric sites where it regulates the expression of neighboring genes (Biroccio et al., 2013; Simonet et al., 2011; Yang et al., 2011; Ye et al., 2014), we searched genome‐wide for TRF2 binding sites in transduced myotubes (TRF2 up‐ or downregulation) using chromatin immunoprecipitation (ChIP) followed by deep sequencing (ChIP‐Seq). Using peak calling tools, we identified putative TRF2 binding sites along with their genomic distribution and compared peaks between myotubes with up‐ or downregulation of TRF2 (Figure S6). Interestingly, some peaks correspond to TRF2‐bound ITSs (interstitial telomeric sequences) previously characterized in cancer cell lines, such as a binding site at the intron of the *HS3ST4* gene (Simonet et al., 2011; Yang et al., 2011), whose expression is regulated by TRF2 (Biroccio et al., 2013). We first validated ChIP‐Seq data by droplet digital PCR (ChIP‐ddPCR) (Robin et al., 2014) at the TRF2‐enriched peak localized within the *HS3ST4* gene (Figure S7a). Next, we generated a list of genes localized within a distance of 100kb from putative TRF2 binding sites and inversely modulated by TRF2 levels (gain and loss; Figure 3a). By crossing position of these genes with ITSs, we produced a list of 53 genes associated with TRF2 and with at least one ITS (Table S1). Among them, the *SIRT3* gene encoding for the mitochondrial Sirtuin‐3 NAD‐dependent deacetylase (SIRT3), which is involved in complex I activity (Ahn et al., 2008; Karamanlidis et al., 2013), mitochondrial homeostasis (Sack, 2012), and senescence (Wiley et al., 2016), represents an attractive and coherent TRF2 target regarding metabolic changes induced by *TERF2* downregulation. Using ChIP‐ddPCR, we confirmed changes in TRF2 binding to the ITSs of the *SIRT3* locus as well as to the *SIRT3* promoter (Figure S7b–c). Upon *TERF2* downregulation, these binding sites were depleted in TRF2, whereas the *CICp23* subtelomeric pseudogene promoter, which is located closer to the telomere, was enriched. We speculated that TRF2 binding loss triggers large‐scale subtelomeric chromatin changes that expose the *CICp23* gene, which is localized between the 11p telomere and the *SIRT3* locus, to the spreading and relocalization of the shelterin protein complex, an explanation consistent with the dynamic chromatin boundaries described for other telomeres (Koering et al., 2002; Robin et al., 2014).

Next, we examined the expression levels of *SIRT3* and other SIRTs. In postmitotic myotubes but not in dividing myoblasts, *TERF2* depletion led to specific and restricted downregulation of *SIRT3* (Figures 3b and S7), consistent with the presence of TRF2 binding sites in the vicinity of the *SIRT3* gene (Figure S7b–c). Using enriched mitochondrial extracts from transduced human myotubes, we found that SIRT3 deacetylase activity was decreased upon *TERF2* knockdown (Figure 3c). Together, these results show that TRF2 is involved in the direct regulation of *SIRT3* expression and enzymatic activity.

Notably, the expression of *PGC1α*, a mitochondrial regulator gene known to be positively regulated by the RAP1 shelterin subunit (Martínez et al., 2013) and repressed by p53 activation (Sahin et al., 2011), is inversely proportional to *TERF2* expression (myotubes; Figure 3b). In human myotubes, RAP1 levels were not modulated by *TERF2* downregulation (Figure S2c). These results indicate that the mitochondrial dysfunction triggered by TRF2 depletion does not result from a RAP1‐mediated downregulation of *PGC1α* and is consistent with our observation that TRF2 depletion does not activate p53 (Figure S3a–b). Thus, *PGC1α* upregulation is likely a compensatory mechanism of the mitochondrial dysfunction and increased ROS production triggered by TRF2 inhibition rather than directly regulated by shelterin proteins.

Importantly, we asked whether the restoration of a physiological level of SIRT3 in TRF2‐compromised myotubes would rescue the oxidative stress and mitochondrial dysfunction triggered by TRF2 depletion. Impressively enough, upon *TERF2* knockdown, ectopic *SIRT3* expression (Figures 3d and S7e) restored SIRT3 activity (Figure 3f), mitochondrial activity (Figure 3e, sh*TERF2 *vs. Rescue,* p* = .0337; Figure 3f, sh*TERF2 *vs. Rescue,* p* < .0001; Figure S7f), decreased ROS production (Figures 3g and S8a), and mitochondrial DNA content (Figure S8b). These results demonstrate that *SIRT3* repression in myotubes is responsible for the changes in mitochondrial respiration and elevated ROS levels triggered by TRF2 downregulation.

### 2.5 TRF2 created a long‐distance subtelomeric loop involving the SIRT3 locus

**Figure 4:**
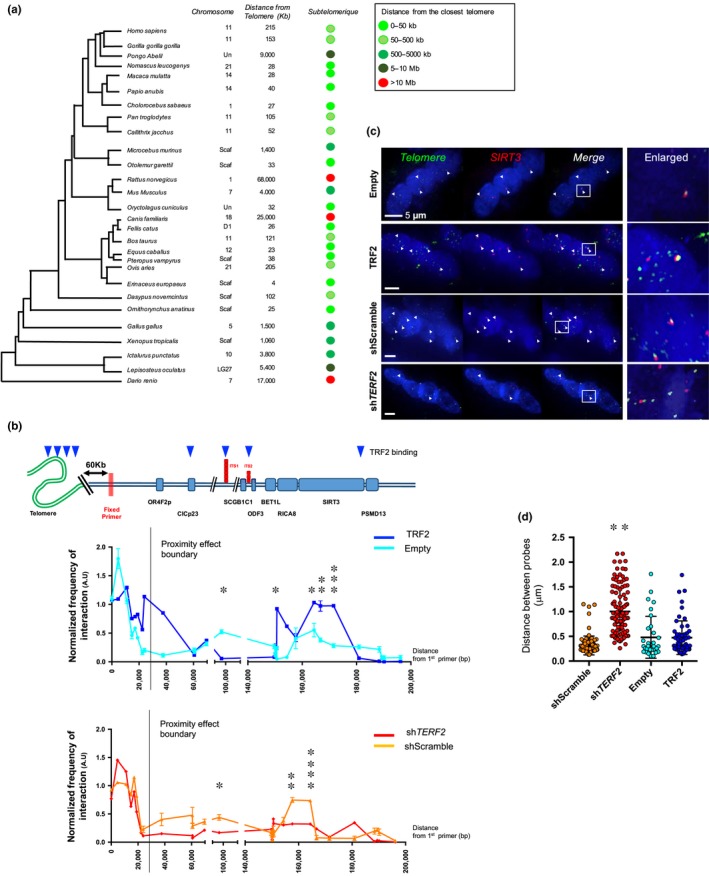
TRF2 depletion modifies higher‐order conformation of the subtelomeric *SIRT3* gene. (a) Phylogenetic tree and *SIRT3* localization through evolution. For each species, we report the chromosomal position when available (Scaf: scaffold genome; Un: unplaced) and deducted distance from the closest telomere. Remarkably, the subtelomeric position of *SIRT3* is well conserved, more particularly among mammals and primates. (b) Chromatin conformation capture (3C) assay. Schematic representation of the 11p locus with TRF2 detected binding regions (top); genes and primer localization are aligned to the 3C performed on the 11p locus (first 2Mb), reported below. Myotubes were collected 10 days after transduction. Each measure represents the amplification of interactions involving a fixed primer (red bars) and a second primer along the 2Mb of the locus, both located in proximity of a *Hind*III restriction site. *TERF2* overexpression enhances subtelomeric looping and interaction between the distal part of the 11p subtelomere and the *SIRT3* locus (Empty vs. TRF2 position: 164,360, *p* = .0195; 166,712, *p* = .0039; 171,528, *p* = .0008; unpaired *t* test; α = 0.05), whereas *TERF2* downregulation decreases these interactions (shScramble vs. sh*TERF2* position: 157,640, *p* = .0017; 164,360, *p* < .0001; 166,712, *p* = .074; 171,528, *p* = .4785; unpaired *t* test; α = 0.05). *N* = 6 per data point (technical duplicates of biological triplicates), means ± *SD* are shown. No difference was detected between control conditions (Figure S7). (c–d) DNA FISH in 3D‐preserved transduced human myotubes and associated quantifications (c). Telomeres were labeled in green and the *SIRT3* locus in red. Distances between gravity centers of the *SIRT3* locus signal and the closest telomeric signal are reported; *N* > 40 nuclei per condition, means ± *SEM* are shown. Single nucleus cells were excluded from the analysis (e.g., less than 5% of total values). We observe a significant increase in separated signals corresponding to an increased distance between the telomere and *SIRT3* locus in myotubes transduced with sh*TERF2* (Kruskal–Wallis multiple comparisons test; α = 0.05) compared to the other conditions (shScramble, Empty, TRF2). *p < .05; **p < .01; ***p < .001

Given the subtelomeric conserved localization of the *SIRT3* gene at a distance of approximately 200 kb from the 11p telomere in human cells (Figure 4a), and the fact that its neighboring genes are not affected by *TERF2* downregulation (Figure S9a), we hypothesized that *SIRT3* could be regulated by a discontinuous telomeric position effect (Koering et al., 2002; Robin et al., 2014) involving a subtelomeric chromatin loop (Lebrun, Fourel, Defossez, & Gilson, 2003; Wood et al., 2014). We applied chromosome conformation capture (3C) and three‐dimensional (3D) DNA fluorescent in situ hybridization (FISH) in our transduced myotube model. Using 3C, we identified a TRF2‐dependent chromatin loop encompassing the 11p telomere and the *SIRT3* locus (Figure 4b). This loop was observed less frequently in TRF2‐compromised myotubes (position 164,360, *p* < .0001) and more frequently in TRF2‐overexpressing myotubes (position 164,360, *p* = .0195), indicating that the formation of this loop is dependent on TRF2 level. No difference was observed between control conditions (shScramble vs. Empty; *p* > .1; Figure S9b). Using specific probes against telomeres and the *SIRT3* locus, we further confirmed by 3D DNA FISH that this chromatin loop is less frequent in the nuclei of myotubes transduced with sh*TERF2* constructs, but conserved under upregulation conditions (Figure 4c–d).

Given the striking conservation of subtelomeric localization of the *SIRT3* locus across evolution (Figure 4a), we next explored whether the ability of TRF2 to mediate a large SIRT3 subtelomeric loop was conserved in other species. We monitored the formation of the loop and Trf2 dependence in mitotically arrested mouse embryonic fibroblasts (MEFs treated with mitomycin). In mice, *Sirt3* is localized 4 Mb from the 7q chromosome, allowing for the use of a unique 7q telomeric probe. Using 3D‐FISH, we observed an interaction between the *Sirt3* locus and the 7q telomeric probe. As in human myofibers, the loop was less frequent upon *Terf2* knockdown (Figure S9c–d) confirming the role of TRF2 in the transcriptional regulation of the *SIRT3* locus.

### 2.6 Terf2 gene ablation in skeletal muscle increases the proportion of oxidative fibers and leads to mitochondrial changes

**Figure 5:**
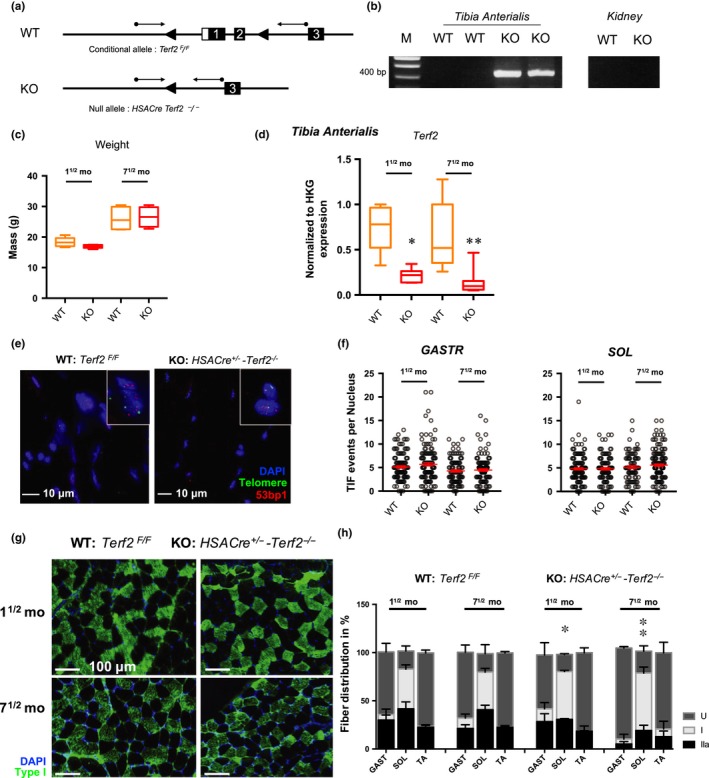
Muscle‐specific *Terf2*‐deficient mice do not trigger telomere damages but display increased oxidative fibers. (a) Scheme depicting the *Terf2* locus within the targeted conditional allele (*Terf2^F^*/*^F^*) and the null allele (*Terf2^−^/^−^*). PCR primers used for genotyping are indicated by arrows. (b) PCR analysis of transgenic mice. Muscle‐specific Terf2 KO allows the amplification of a 385 bp PCR product in muscle fibers. M: molecular weight markers; WT: *Terf2^F^/^F^* mice, KO: *HSACre‐Terf2^−^/^−^*. (c) No statistical differences of weight were seen among mice of the same age. (d) Knockdown of *Terf2* in mature muscle fibers was further validated in RT‐qPCR and IFs (Figure S10). *Terf2* expression quantified by RT‐qPCR in RNA extracted from skeletal muscle (TA) from WT and KO mice. Each measure represents the average fold‐change expression of eight independent repetitions (four mice in technical duplicate) normalized to three housekeeping genes (*Hprt*, *Ppib*, and *Gapdh*; ΔΔCt method). Means ± *SEM* with associated statistical significance are reported (Kruskal–Wallis multiple comparisons test; α = 0.05). € Telomeric induced focus (TIF) analysis in transgenic WT and KO mice and associated quantification reported in (f) in the *soleus* (*SOL*) and *gastrocnemius* (*GASTR*). For each group, we report the number of TIFs counted in 30 nuclei per mice for a minimum of four mice (minimum of 120 nuclei per group). Means ± *SEM* are shown. No statistical differences were observed between groups (Kruskal–Wallis multiple comparisons test; *α* = 0.05). (g–h) Fiber‐type characterization in *HSACre^+^/^−^‐Terf2^−^/^−^* mice using myosin heavy chain antibodies against type I and IIa fibers for the *gastrocnemius* (GAST), *soleus* (SOL), and *tibia anterialis* (TA). *n* = 5 mice per group. Undetermined fibers (U) represent fibers that remained unstained after using both antibodies (e.g., against type I and type IIa fibers, Figure S10). Means ± *SEM* are shown. * <.05; ** <.001; *** <.0001

**Figure 6:**
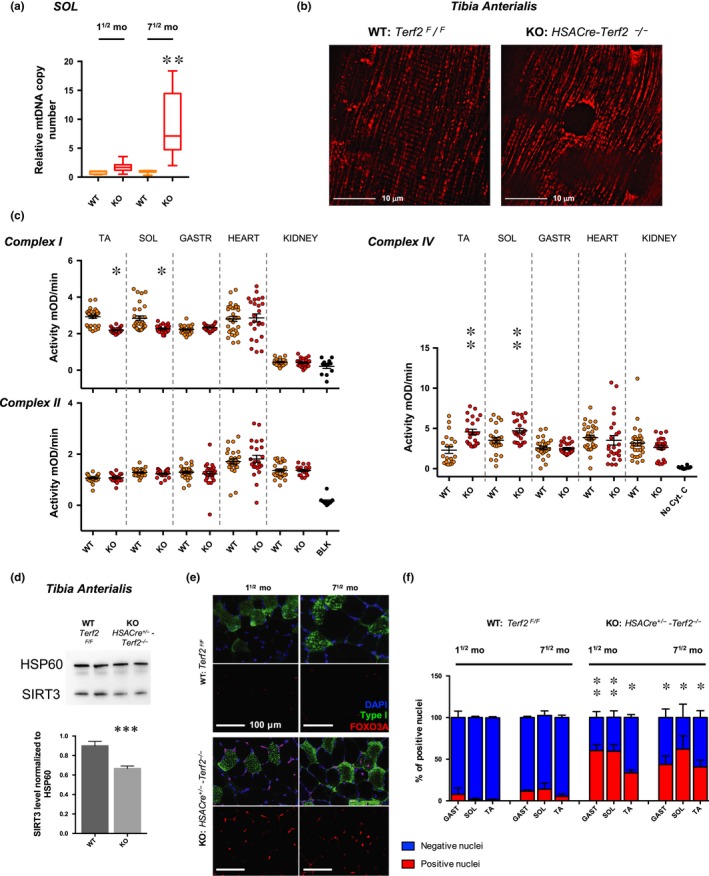
Muscle‐specific *Terf2*‐deficient mice exhibit increased mitochondrial DNA content, mitochondrial dysfunction, and nuclear Foxo3a accumulation. (a) Relative quantification of mitochondrial DNA content in the *soleus* of transgenic mice (WT and KO). *Terf2* KO mice exhibit a significantly higher mtDNA content (Kruskal–Wallis multiple comparisons test; *α* = 0.05). *n* = 12 per group, means ± *SEM* are shown. (b) Electroporated *tibia anterialis* (TA), using Mito‐DsRed in control and transgenic mice (*n* = 4 per group). Electroporation of the construct allows one to stain the mitochondrial network in tissues. The mitochondrial network of KO mice (right) appears punctuated and less structured as in controls (left). (c) Mitochondrial complex I, II, and IV activity in tissues (e.g., kidney; heart; *soleus*; *tibia anterialis*) from 40‐week‐old transgenic WT and KO mouse (*n* = 10 per data point). *Terf2* KO mice exhibit skeletal muscle‐specific mitochondrial defects. We report a decreased complex I activity (*p* < .05, paired, two‐tailed Student's *t* test; *α* = 0.05) and an increased complex IV activity (*p* < .001, paired, two‐tailed Student's *t* test; *α* = 0.05), potentially part of a compensating phenomenon. No statistical differences were observed between WT and KO kidney and heart extracts. (d) SIRT3 immunoblots of enriched mitochondria extracts from the *tibia anterialis* (TA) of transgenic mice using HSP60 as loading control. *N* = 5 per group. *Terf2* abolished expression results in decreased SIRT3 level (WT vs. KO, *p* = .0007). (e–f) Foxo3a immunofluorescence and associated quantifications in muscles along with type I staining (e.g., GAST, SOL, and TA). No statistical association was found among positive nuclei and fiber types. *Terf2* KO mice exhibit a higher percentage of Foxo3a‐positive nuclei (aged and muscle‐matched KO vs. WT, *p* < .05; Kruskal–Wallis multiple comparisons test; α = 0.05). * <.05; ** <.01; *** <.001

To generalize the results obtained with human myotubes, we explored the role of Trf2 in mouse skeletal muscle mitochondria by generating mice where *Terf2* is specifically abrogated in mature fibers (*HSACre‐Terf2*, Figure 5a5b). We confirmed Trf2 depletion in skeletal muscle nuclei (Figure S10) and *Terf2* downregulation in whole muscle biopsies (Figure 5d). Up to the age of 10 months, these mice exhibit apparent normal development, weight (Figure 5c), and behavior. Akin to human myotube results, transgenic mouse skeletal muscles (e.g., *gastrocnemius*, *soleus*) did not exhibit any increase in TIFs (Figure 5f–g) but showed an increased proportion of oxidative fibers in the *Soleus* as compared to controls (Figures 5h and S10b, *p* = .037, *p* = .0173 at 1^½^ and 7^½^ months, respectively). Then, we set out to address whether this metabolic shift was associated with mitochondrial changes. By qPCR, we found a higher level of mitochondrial DNA in transgenic mice (Figure 6a) along with a disturbed mitochondrial network represented by less grid‐like structures (Figure 6b). To strengthen these observations, we tested the mitochondrial complex activities in skeletal muscles (*soleus*, *tibia anterialis*,* gastrocnemius*), and in control tissues (Heart, Kidney). We found a modified complex I and IV activity, decreased and increased, respectively (Figures 6c and S11), restricted to skeletal muscle tissues (e.g., *soleus* and *tibia anterialis*) and without modification of complex II. As proposed for human myotubes, the increased activity of complex IV could constitute a compensatory mechanism of complex I dysfunction leading to oxidative stress (Kotiadis et al., 2014). Once again consistent with the results obtained with human myotubes, we detected a decrease of *Sirt3* expression in enriched mitochondrial extracts and a potent activation of Foxo3a in skeletal muscle of transgenic mice (Figure 6d6f).

Altogether, these mouse in vivo results confirm the role of the TRF2‐SIRT3 axis in skeletal muscle mitochondrial functions revealed in vitro in human myotubes.

## 3 DISCUSSION

Our results revealed a downregulation of TRF2 in a representative cohort of human skeletal muscle biopsies (*n* = 20) over lifetime (Figure 1). The same muscle samples exhibit progressive telomere shortening with age, validating the significance of our cohort regarding the previously observed telomere shortening during muscle aging (Daniali et al., 2013). Mimicking this in vivo regulation, TRF2 downregulation in a fully controlled in vitro human myotube model did not trigger telomere deprotection but instead triggered mitochondrial changes reminiscent of those observed during development and aging, that is, increased ROS and oxidative metabolism (Figure 2). Moreover, we report a close overlap of our in vitro observations in vivo, using a transgenic mouse model of skeletal muscle‐specific *Terf2* ablation. In both models, downregulation of *TERF2* triggered an increase of FOXO3A to the nucleus (Figures 2b and 6e). A finding that is in agreement with previous work describing FOXO3A as a stress sensor (Milan et al., 2015; Sandri et al., 2004) and mitochondrial regulator (Ferber et al., 2012). Hence, knowing that FOXO3A function declines with age (Mueller et al., 2014) and is linked to longevity (Albani et al., 2014; Flachsbart et al., 2009), one can hypothesize that upregulation of FOXO3A detected in our models (e.g., myotubes, transgenic mice) illustrates an age‐dependent protective mechanism.

Based on these results, we propose that the TRF2 downregulation, starting in early adulthood as a developmentally regulated process, contributes to the physiological adaptation and oxidative metabolic changes occurring in muscles over lifetime (Lexell, 1995; Miljkovic et al., 2015; Powers, Radak, & Ji, 2016). An interesting mechanism would be a direct regulation of *TERF2* by muscle‐specific factors involved in fiber‐type switching (Murgia et al., 2017) or through changes in the Wnt and beta‐catenin pathways that regulate muscle differentiation but also involved in *TERF2* regulation (Diala et al., 2013).

Reminiscent of the antagonistic pleiotropic models of telomeric DNA shortening (Harley, 1997), TRF2 downregulation might be beneficial at young ages and detrimental for muscle function at older ages. Consistent with this hypothesis, a depletion of TRF2 in mouse skeletal muscle triggers a modification of fiber‐type proportion toward an increased oxidative metabolism allude for a premature aging phenotype (Alnaqeeb & Goldspink, 1987; Lexell, 1995; Miljkovic et al., 2015; Verdijk et al., 2007). Beyond the scope of this study, further characterization of this transgenic model will allow one to appreciate the contribution of TRF2 in the changes and adaptation of skeletal muscle metabolism and physiology during development and aging.

At the molecular level, we elucidated a mechanism by which TRF2 alters muscle cell mitochondrial oxidative respiration. We showed that TRF2 positively regulates the expression of *SIRT3*, a key mitochondrial gene associated with human longevity (Albani et al., 2014; Flachsbart et al., 2009)^,^ and SIRT3‐dependent deacetylase activity (Figure 3). As described by others, SIRT3 is a crucial regulator of basal physiology (Ansari et al., 2017) and affects human life span (Kincaid & Bossy‐Wetzel, 2013). Importantly, restoring physiological SIRT3 levels in TRF2‐compromised myotubes rescued the mitochondrial phenotype and decreased ROS levels to those of control cells, establishing that a TRF2‐SIRT3 axis modulates the oxidative metabolism in skeletal muscle fibers.

TRF2 appears to be directly involved in *SIRT3* transcriptional regulation by binding to several sites around the subtelomeric *SIRT3* locus and mediating the formation of a long‐distance subtelomeric chromatin loop between the *SIRT3* locus and its proximal telomere (Figure 4). The subtelomeric location of the *SIRT3* locus is highly conserved throughout evolution. Remarkably, a similar Trf2‐dependant long‐range loop between the *Sirt3* locus and the telomere is also observed in mouse mitotically arrested cells (Figure S9) and Trf2 ablation in mouse skeletal muscle leads to Sirt3 downregulation, highlighting the functional importance of the TRF2‐mediated chromatin loop in regulating *SIRT3* gene expression and, subsequently, oxidative metabolism.

In contrast to the subtelomeric loops encompassing the *TERT* gene at chromosome 5p (Kim et al., 2016) and the *SORBS2* gene at the 4q35 locus (Robin, Ludlow, et al., 2015), the telomere‐SIRT3 loop discovered in this study is required for gene activation as previously observed (*TEAD4*; Robin et al., 2014). The underlying mechanism linking subtelomeric loops to gene regulation and the peculiar telomere protection at play in long‐lived postmitotic cells are now key questions for future studies. Whether telomere shortening concomitant to TRF2 downregulation in skeletal muscle destabilizes the telomere‐*SIRT3* loop, as observed for other loci (Kim et al., 2016; Robin, Ludlow, et al., 2015; Robin et al., 2014), is an interesting hypothesis. Further, if telomere shortens in human tissues, including skeletal muscle (Daniali et al., 2013), diminution of TRF2, as already reported through lifetime in animal models (e.g., mice, zebrafish; Wagner et al., 2017), remains to be established in other human tissues. Noteworthy, recent studies focusing on another postmitotic tissue (e.g., heart) also link telomere length and TRF2 level to metabolic changes (Chang et al., 2018, 2016; Oh et al., 2003). Last, using a proliferative model (e.g., transduced myoblasts), we observed an increase of the DDR pathway (Figure 1) but no changes in *SIRT3* gene expression (Figure S7d), further arguing in favor of a telomere‐dependent mechanism restricted to postmitotic tissues.

Overall, our work uncovers a TRF2‐SIRT3 axis that connects telomere changes to muscle adaptative metabolism, development, and aging. This finding holds the potential to drive future research on physiological and pathological changes occurring during the lifetime in muscles and other postmitotic tissues (e.g., brain, bones, or fat among others).

## 4 METHODS

Detailed methods and Supplemental Material can be found with this article online.

## CONFLICT OF INTEREST

None declared.

## AUTHOR CONTRIBUTIONS

J.D.R. executed the experiments, helped design, and wrote the manuscript. L.S. and V.R. helped design. M.S.J.B., L.L, W.L., and C.L. provided technical help; H.P., F.T., E.P., and O.C. assisted and performed the bioinformatics analysis of the ChIP‐Seq results. F.M. and S.S. obtained muscle biopsies. J.Y. helped project coordination. E.G. designed the project and wrote the manuscript. All authors edited the manuscript.

## Data Availability

All original unprocessed data used for this study are available through a Mendeley database link provided below. The depository includes all raw data for the building of all figures provides and raw files. We also have included a report summary file. This details the software (Prism/IMARIS) and script (ChIP‐Seq) used for this study as well as specific reagents (human samples, mice, and antibodies) used to generate our results. All information is accessible at: http://dx.doi.org/10.17632/mtst962jfr.1. ChIP‐Seq raw data and analysis have been deposited to GEO database under the accessing number http://www.ncbi.nlm.nih.gov/geo/query/acc.cgi?acc=GSE88983. Queries regarding reagents and materials can be addressed to the corresponding authors.
