# Supplementary material for: Mitochondrial function in skeletal myofibers is controlled by a TRF2‐SIRT3 axis over lifetime
Source: Aging Cell. 2020 Jan 28;19(3):e13097. doi: 10.1111/acel.13097 (PMC7059141; doi:10.1111/acel.13097)
Supplement: Supplementary file 1 [file ACEL-19-e13097-s001.pdf]

Figure S1

a

PATIENT	SEX	AGE (At Biopsy)		LOCALIZATION
1	♂	14w	Fetus	QUADRICEPS
2	♂	22w	Fetus	QUADRICEPS
3	♂	22w	Fetus	QUADRICEPS
4	♂	37w	Fetus	QUADRICEPS
5	♂	37w	Fetus	QUADRICEPS
6	♂	17	Teenager	LEFT DELTOÏD
7	♂	17	Teenager	QUADRICEPS
8	♀	18	Teenager	LEFT DELTOÏD
9	♂	18	Teenager	QUADRICEPS
10	♀	19	Teenager	LEFT DELTOÏD
11	♂	24	Adult	LEFT DELTOÏD
12	♀	27	Adult	LEFT DELTOÏD
13	♂	35	Adult	QUADRICEPS
14	♀	60	Elder	RIGHT BICEPS
15	♂	65	Elder	LEFT DELTOÏD
16	♂	68	Elder	QUADRICEPS
17	♂	69	Elder	LEFT BICEPS
18	♂	71	Elder	QUADRICEPS
19	♂	71	Elder	QUADRICEPS
20	♂	72	Elder	QUADRICEPS

b

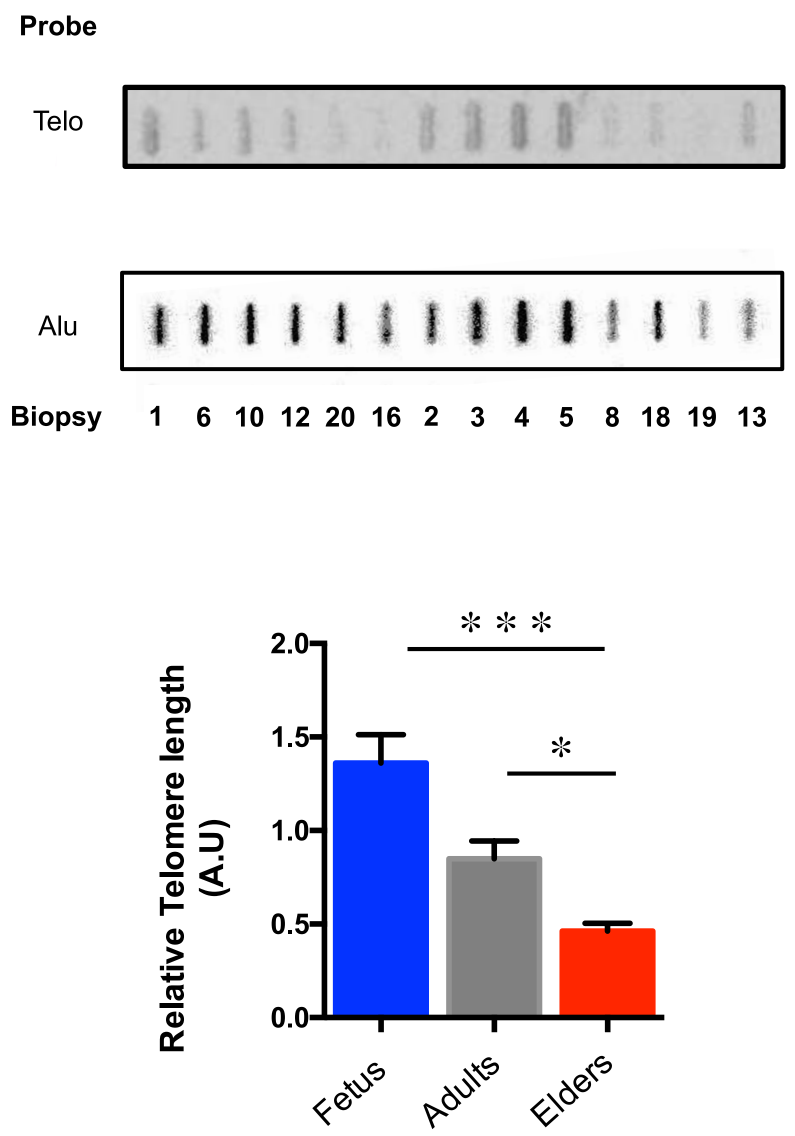

c

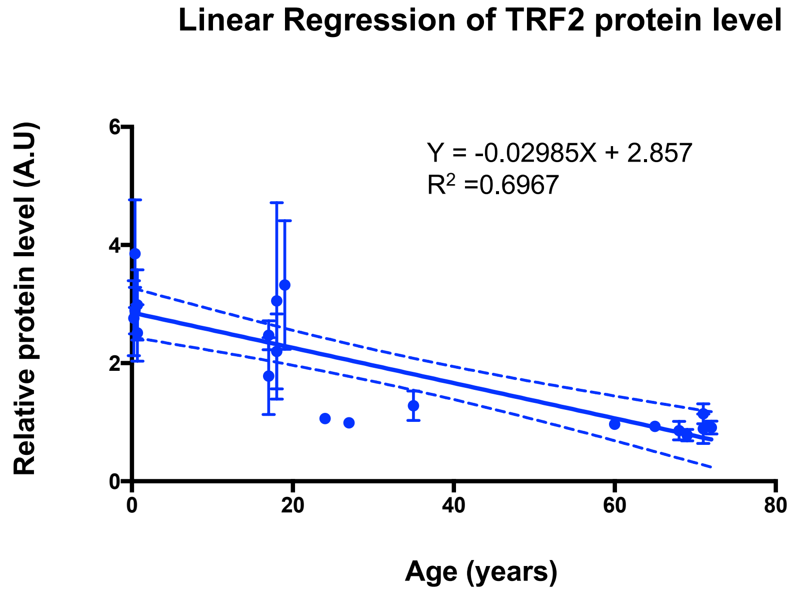

d

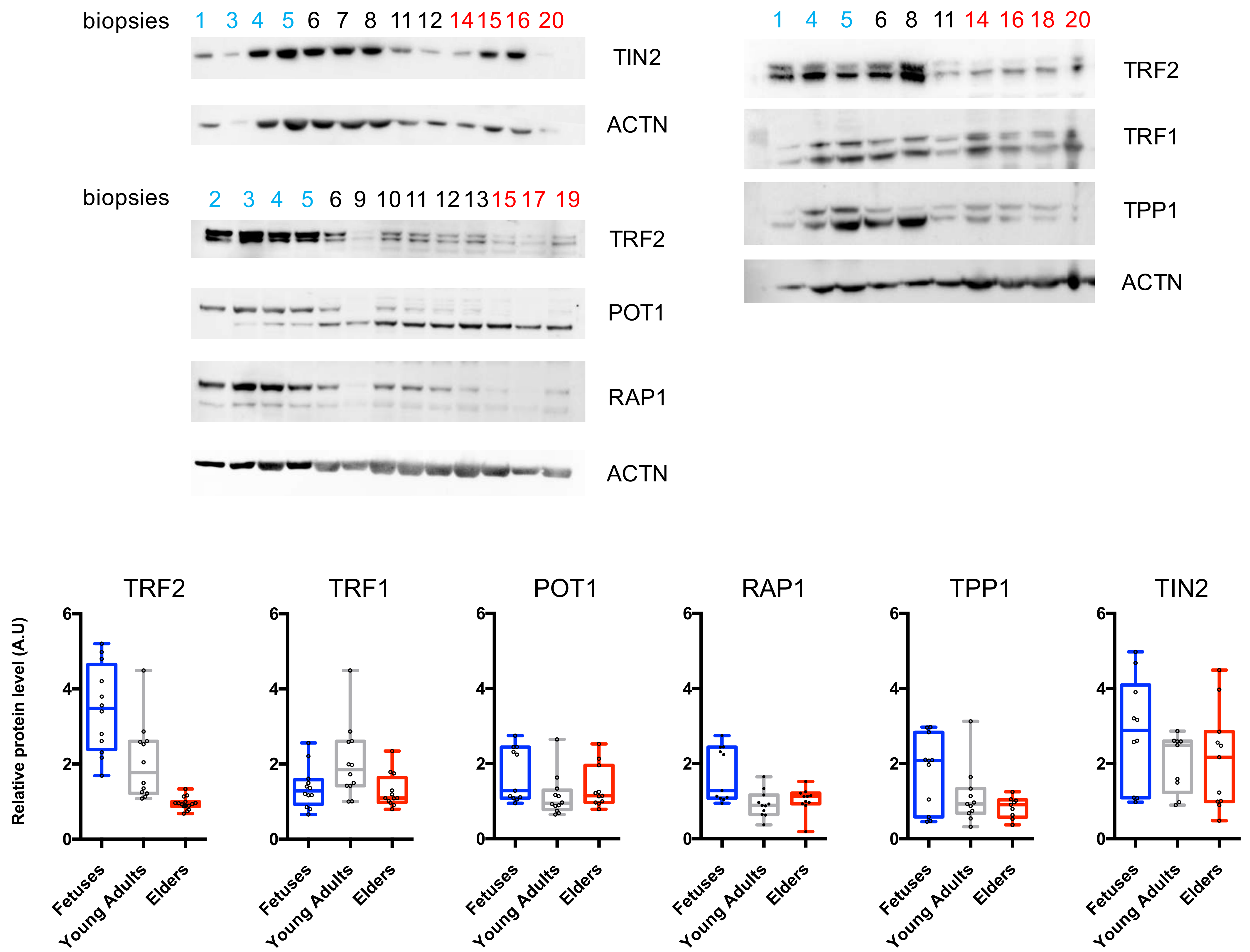

Figure S2

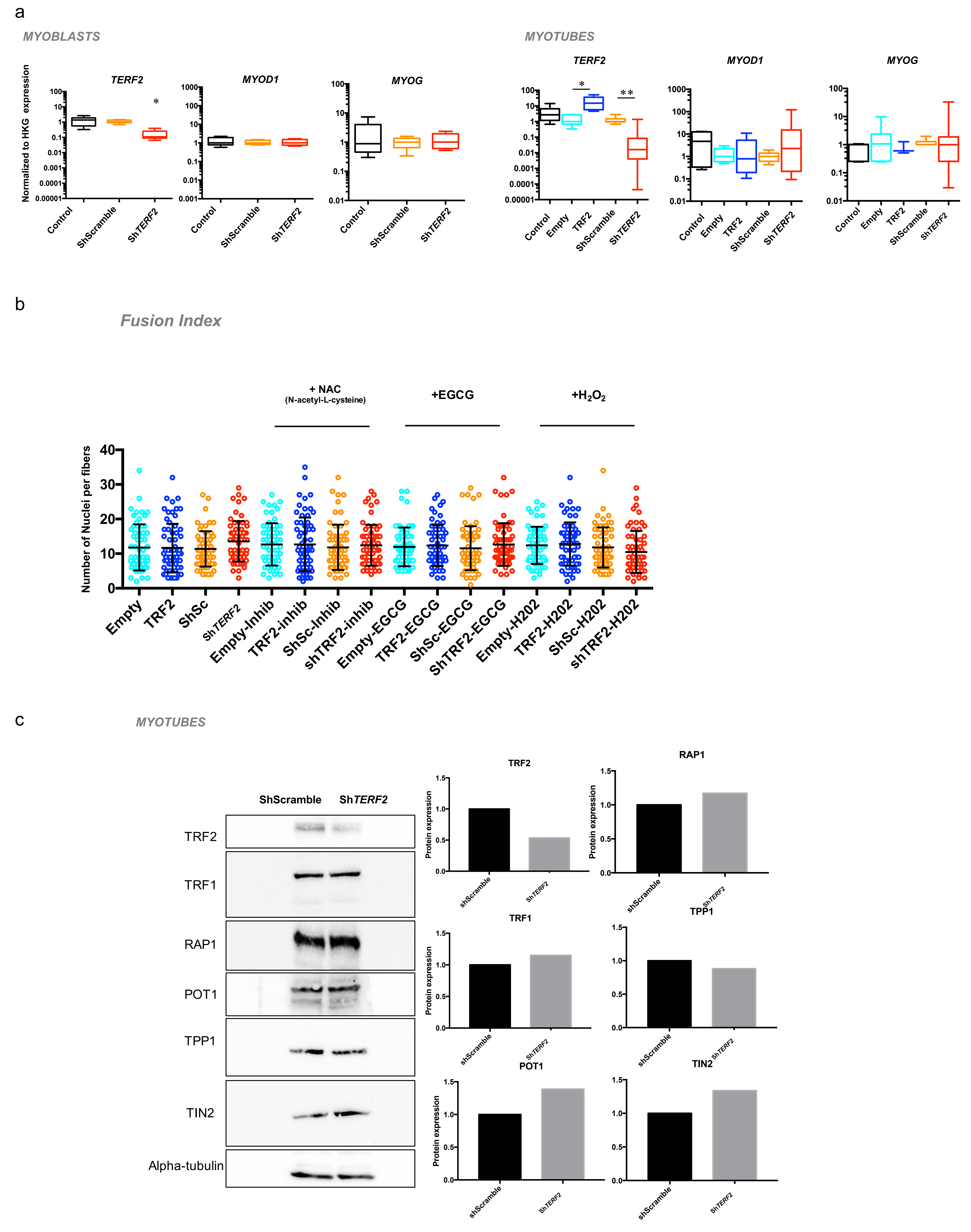
a
MYOBLASTS
MYOTUBES
TERF2
MYOD1
MYOG
Normalized to HKG expression
Control
ShScramble
ShTERF2
Empty
TRF2
b
Fusion Index
+ NAC
(N-acetyl-L-cysteine)
+EGCG
+H2O2
Number of Nuclei per fibers
Empty
TRF2
ShSc
Sh TERF2
Empty-Inhib
TRF2-inhib
ShSc-Inhib
shTRF2-inhib
Empty-EGCG
TRF2-EGCG
ShSc-EGCG
ShTRF2-EGCG
Empty-H202
TRF2-H202
ShSc-H202
shTRF2-H202
c
MYOTUBES
ShScramble
ShTERF2
TRF2
TRF1
RAP1
POT1
TPP1
TIN2
Alpha-tubulin
Protein expression
shScramble
ShTERF2

Figure S3

a

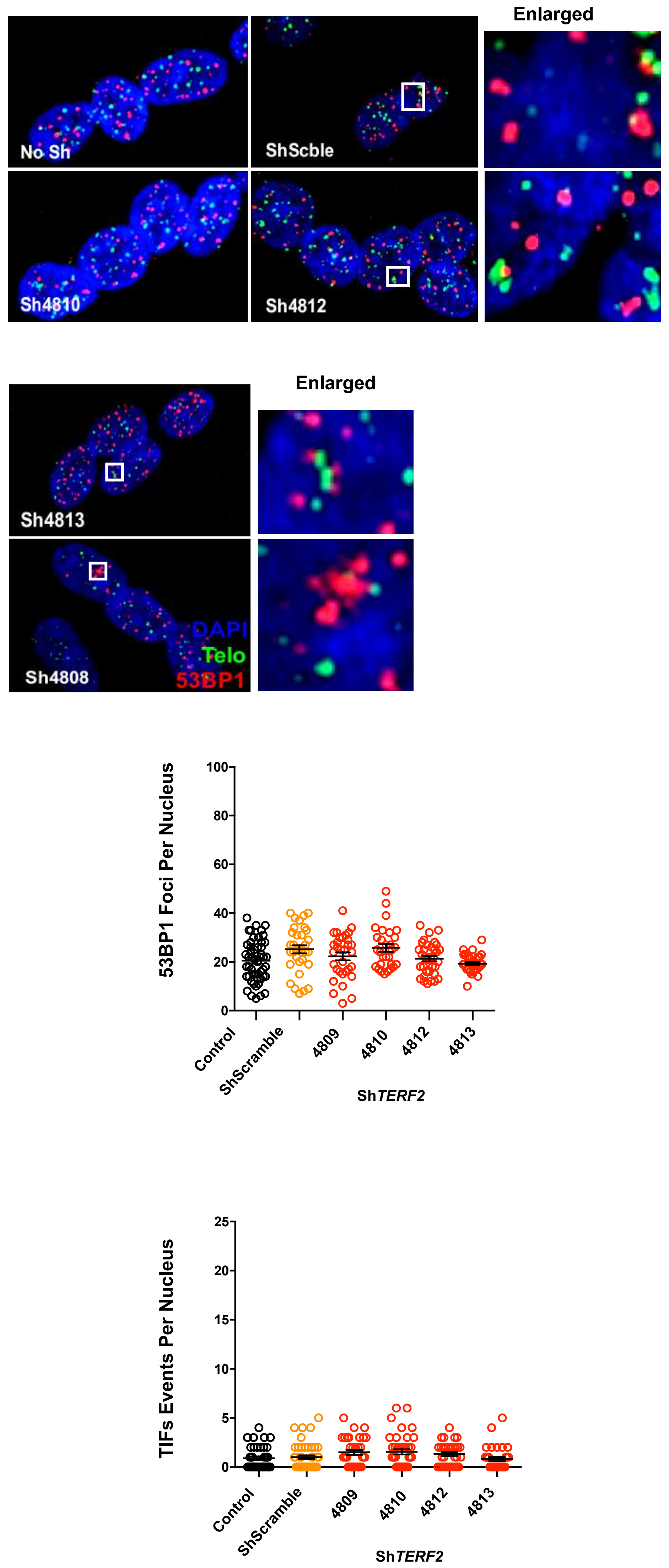

b

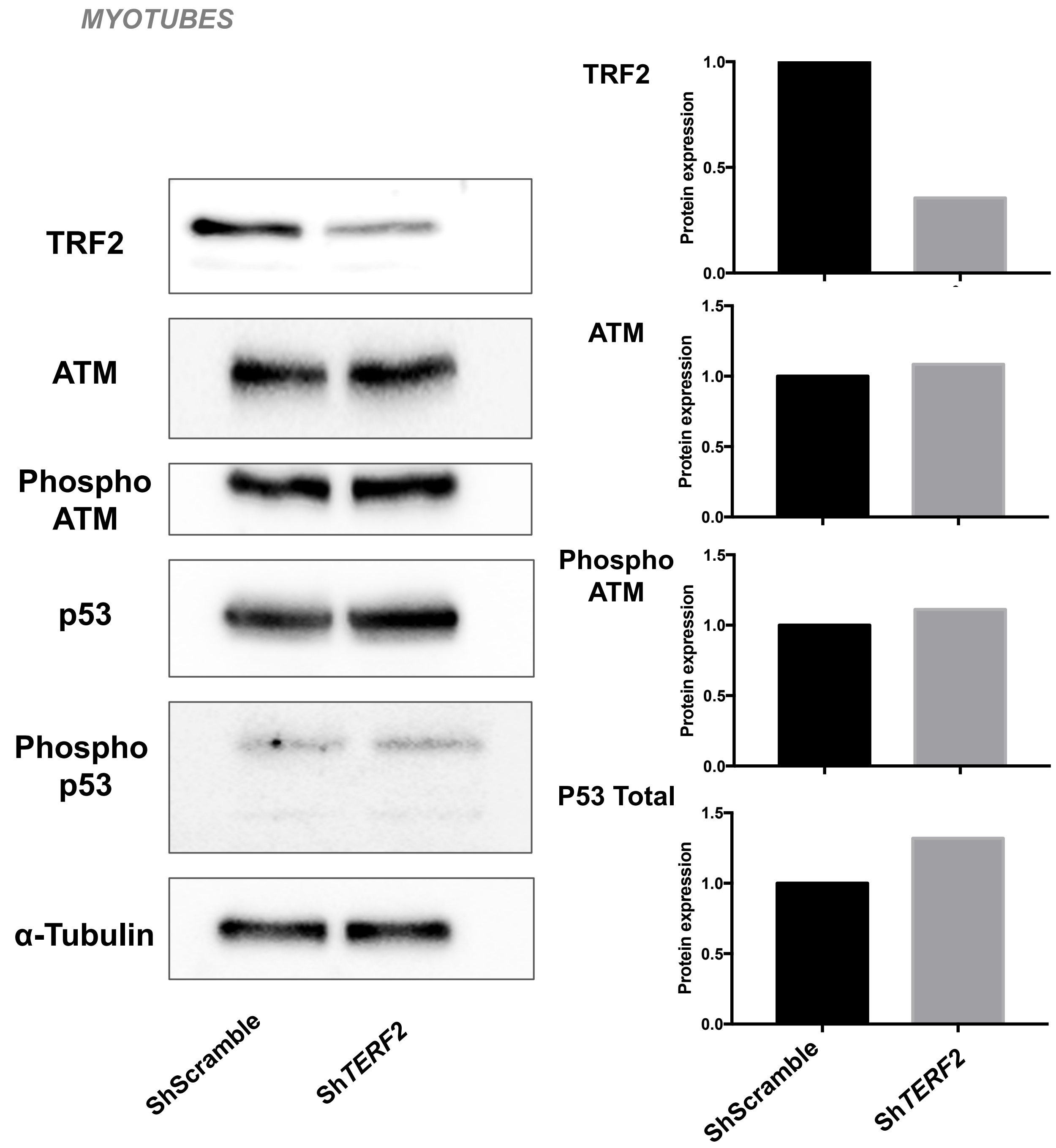

c

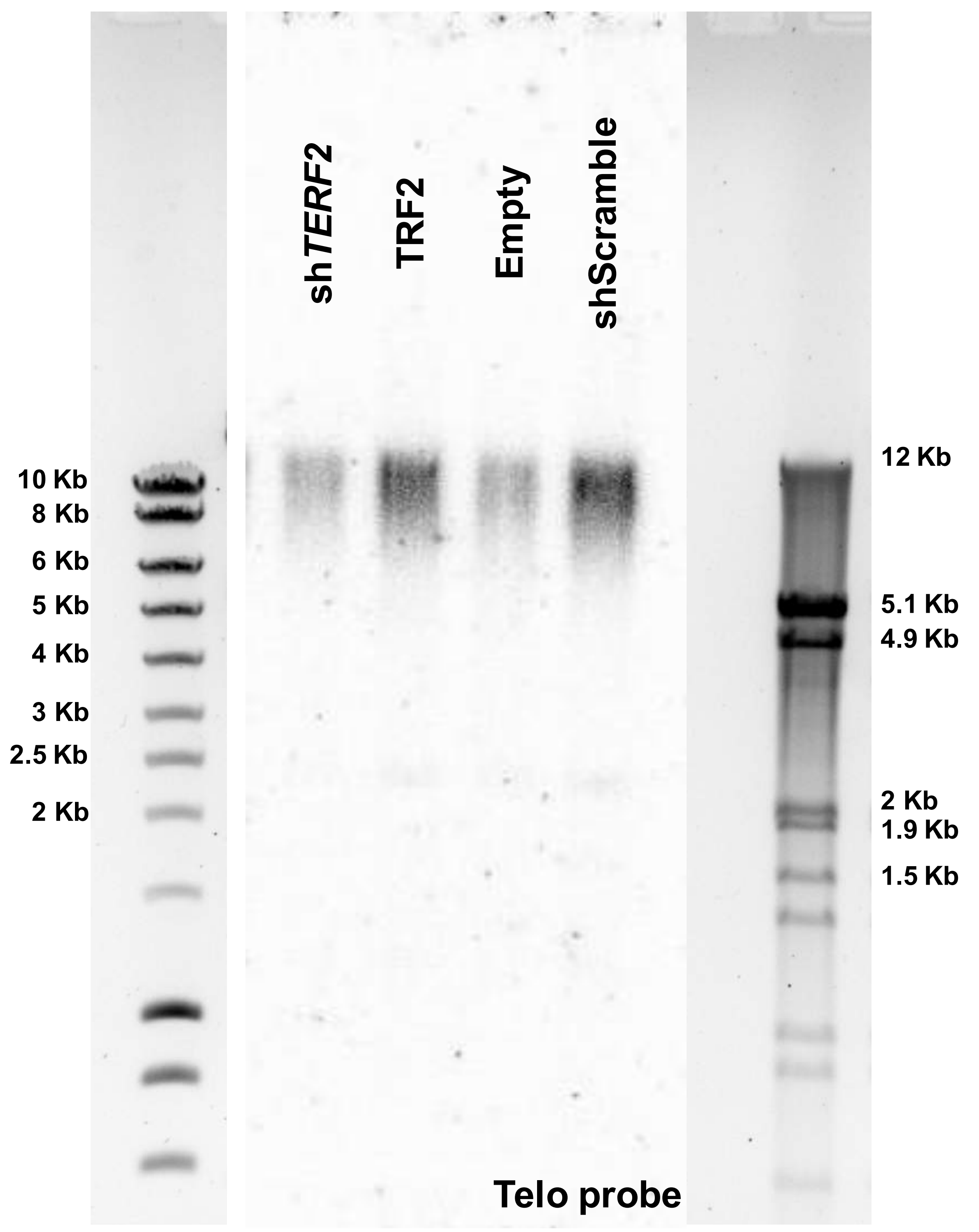

Figure S4

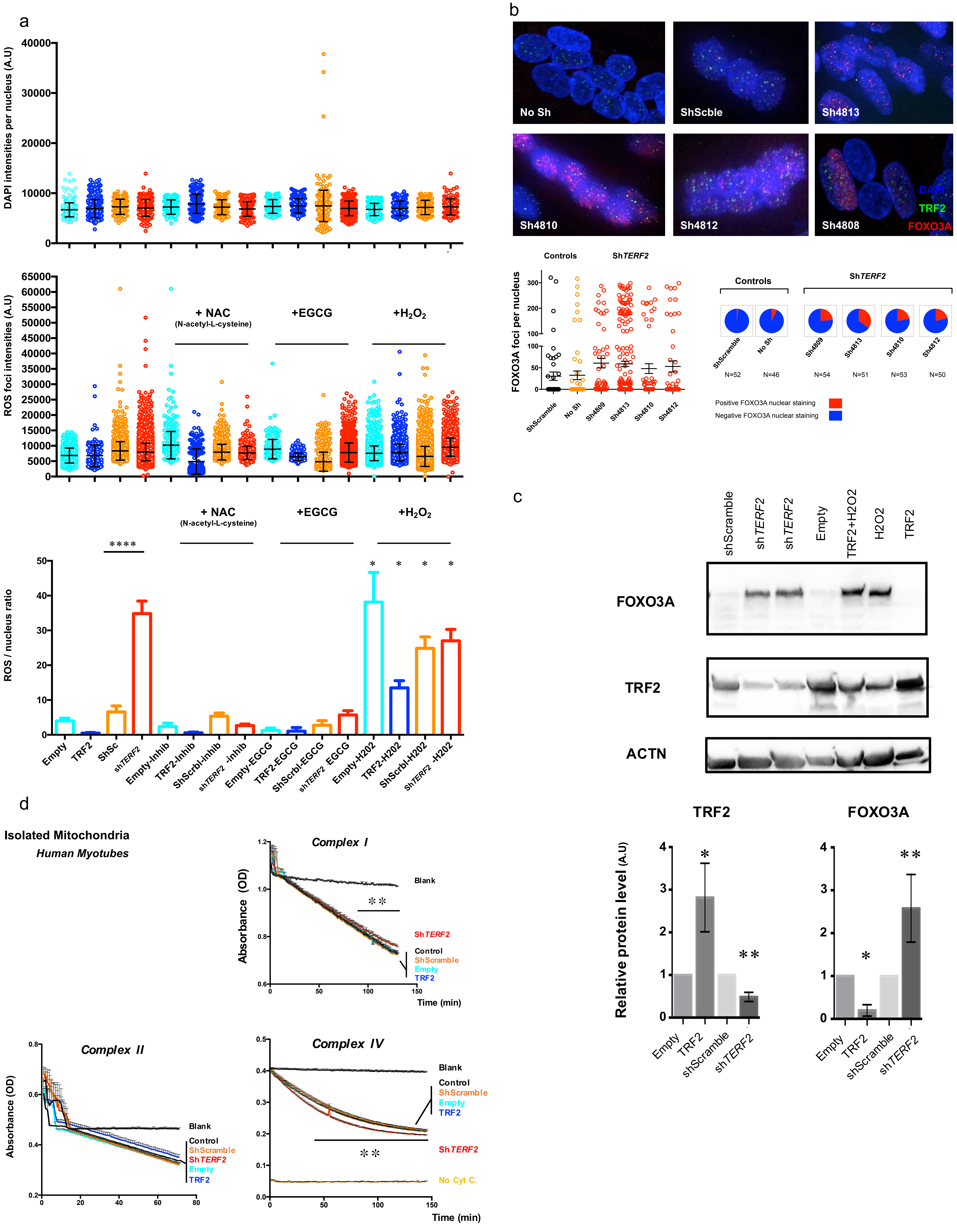
a
DAPI intensities per nucleus (A.U)
ROS foci intensities (A.U)
+ NAC
(N-acetyl-L-cysteine)
+EGCG
+H2O2
ROS / nucleus ratio
****
Empty
TRF2
ShSc
shTERF2
Empty-Inhib
TRF2-Inhib
ShScrbl-inhib
shTERF2 -inhib
Empty-EGCG
TRF2-EGCG
ShScrbl-EGCG
shTERF2 EGCG
Empty-H202
TRF2-H202
ShScrbl-H202
shTERF2 -H202
b
No Sh
ShScble
Sh4813
Sh4810
Sh4812
Sh4808
DAPI
TRF2
FOXO3A
Controls
ShTERF2
FOXO3A foci per nucleus
ShScramble
No Sh
Sh4809
Sh4813
Sh4810
Sh4812
N=52
N=46
N=54
N=51
N=53
N=50
Positive FOXO3A nuclear staining
Negative FOXO3A nuclear staining
c
shScramble
shTERF2
shTERF2
Empty
TRF2+H2O2
H2O2
TRF2
FOXO3A
TRF2
ACTN
TRF2
FOXO3A
Relative protein level (A.U)
Empty
TRF2
shScramble
shTERF2
d
Isolated Mitochondria
Human Myotubes
Complex I
Absorbance (OD)
Blank
ShTERF2
Control
ShScramble
Empty
TRF2
Time (min)
Complex II
Complex IV
No Cyt C.

Figure S5

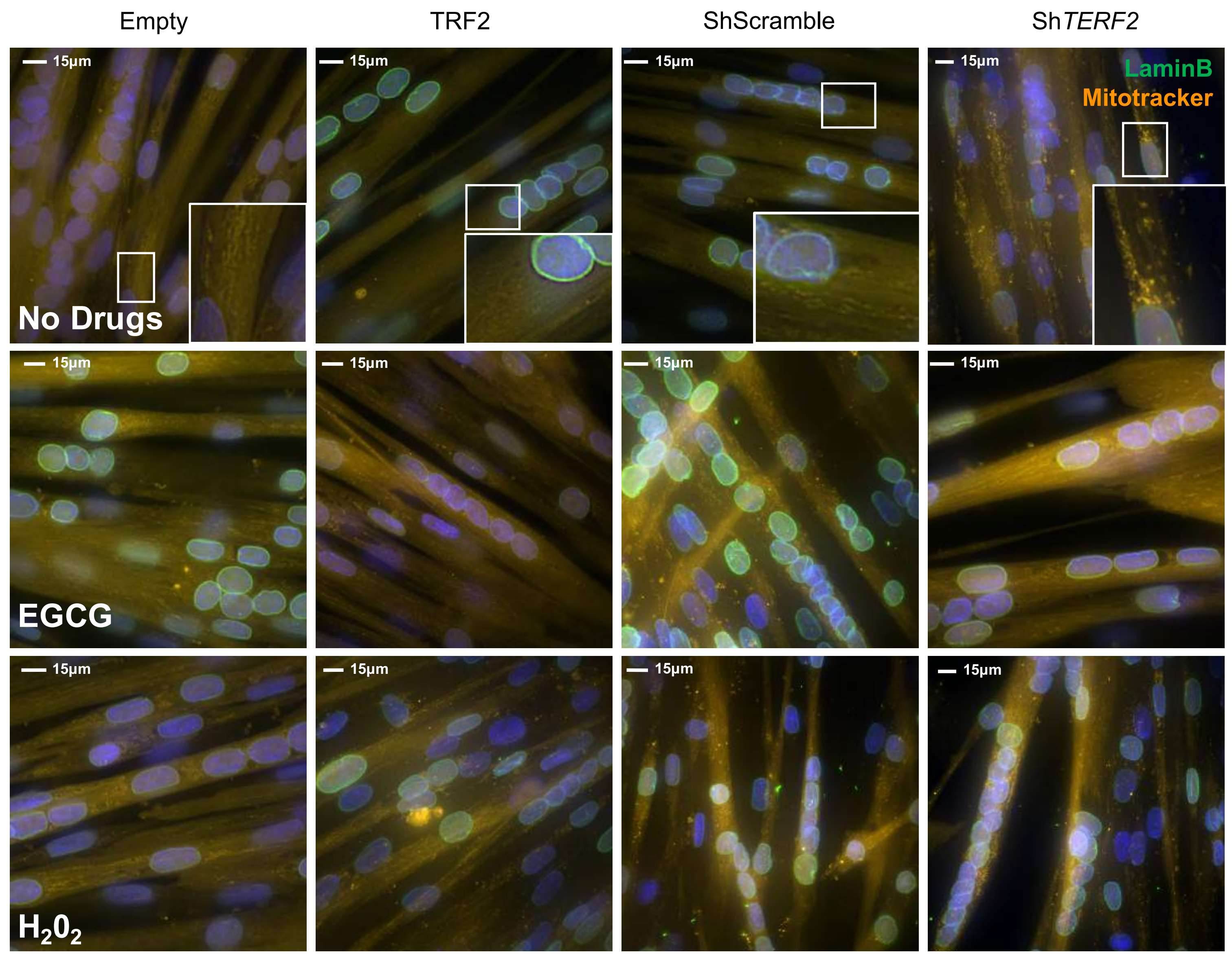

NAD+ / NADH enzymatic staining of transduced myotubes (NADH-TR)

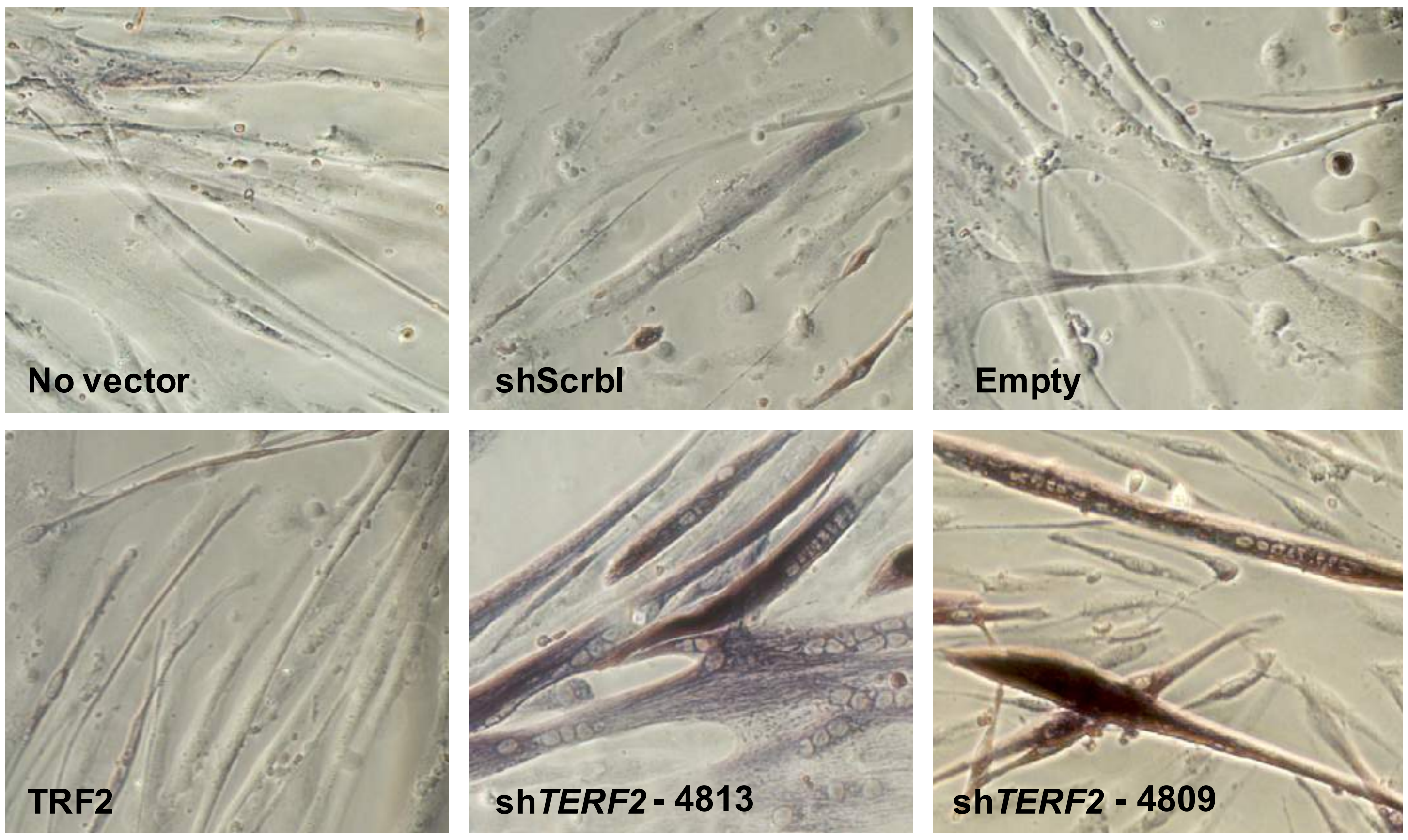

Figure S6

a

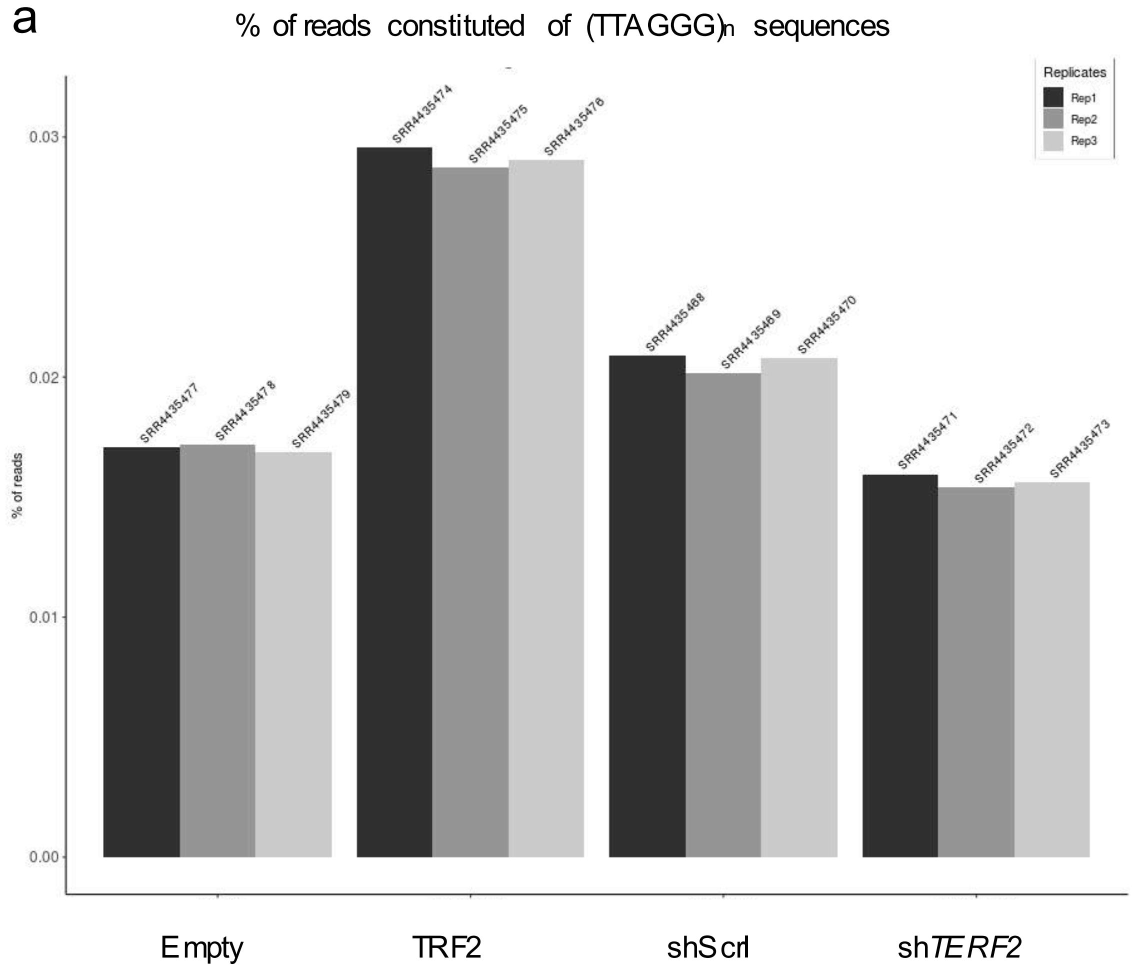

b

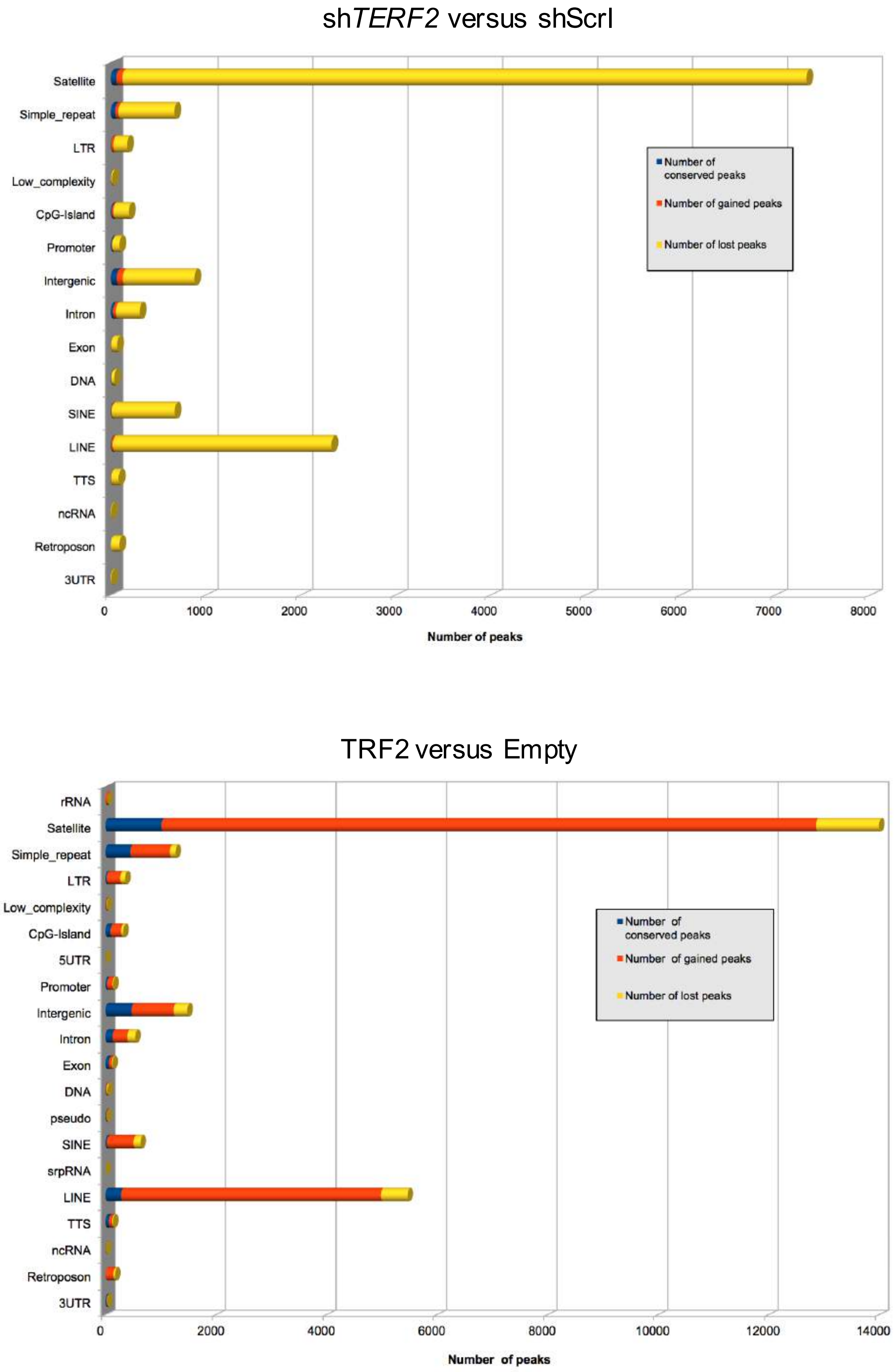

c

Genes loss

Top Canonical Pathways

Name	p-value	Overlap
G-Protein Coupled Receptor Signaling	1,57E-04	13,5 % 38/281
CREB Signaling in Neurons	5,09E-04	13,8 % 30/217
GP6 Signaling Pathway	5,45E-04	15,9 % 21/132
Netrin Signaling	6,98E-04	20,0 % 13/65
cAMP-mediated signaling	8,63E-04	13,4 % 30/224

	GO term	Description	P-value
Biological process	GO:0007186	G protein-coupled receptor signaling pathway	5.69E-9
	GO:0007187	G protein-coupled recep sign path, coupled to cyclic nt second messenger	2.23E-6
	GO:0046777	protein autophosphorylation	5.92E-5
	GO:0007165	signal transduction	8.98E-5
	GO:0007193	adenylate cyclase-inhibiting G protein-coupled recep signaling pathway	2.14E-4
	GO:0007188	adenylate cyclase-modulating G protein-coupled recep signaling pathway	2.32E-4
	GO:0065007	biological regulation	2.91E-4
	GO:0065008	regulation of biological quality	5.24E-4
	GO:0006796	phosphate-containing compound metabolic process	7.14E-4
	GO:0006793	phosphorus metabolic process	7.14E-4
	GO:1901564	organonitrogen compound metabolic process	9.26E-4
	GO:0044248	cellular catabolic process	9.84E-4
Molecular function	GO:0004930	G protein-coupled receptor activity	1.43E-6
	GO:0099528	G protein-coupled neurotransmitter receptor activity	5.04E-5
	GO:0003824	catalytic activity	1.13E-4
Cellular component	GO:0044425	membrane part	1.66E-4
	GO:0005887	integral component of plasma membrane	6.18E-4

Genes gain

Top Canonical Pathways

Name	p-value	Overlap
Leukocyte Extravasation Signaling	3,64E-03	11,1 % 23/208
Netrin Signaling	5,27E-03	15,4 % 10/65
Choline Biosynthesis III	6,04E-03	30,8 % 4/13
Protein Kinase A Signaling	9,42E-03	9,1 % 35/383
Iron homeostasis signaling pathway	1,24E-02	11,5 % 15/131

	GO term	Description	P-value
Biological process	GO:0022610	biological adhesion	4.41E-5
	GO:0007155	cell adhesion	4.41E-5
	GO:0098609	cell-cell adhesion	9.05E-5
	GO:0032879	regulation of localization	1.16E-4
	GO:0030198	extracellular matrix organization	1.41E-4
	GO:0043062	extracellular structure organization	1.41E-4
	GO:0071840	cellular component organization or biogenesis	1.43E-4
	GO:0016043	cellular component organization	1.43E-4
	GO:0033554	cellular response to stress	4.42E-4
	GO:0040012	regulation of locomotion	6.51E-4
	GO:2000145	regulation of cell motility	6.51E-4
	GO:0051894	positive regulation of focal adhesion assembly	7.02E-4
	GO:0010811	positive regulation of cell-substrate adhesion	7.02E-4
	GO:0001954	positive regulation of cell-matrix adhesion	7.02E-4
	GO:1901890	positive regulation of cell junction assembly	7.02E-4
	GO:1903393	positive regulation of adherens junction organization	7.02E-4
	GO:0002376	immune system process	7.67E-4
	GO:0034613	cellular protein localization	7.85E-4
	GO:0070727	cellular macromolecule localization	7.85E-4
	GO:0051270	regulation of cellular component movement	7.85E-4
	GO:0048523	negative regulation of cellular process	8.88E-4
	GO:0050789	regulation of biological process	9.42E-4
Molecular function	GO:0031267	small GTPase binding	3.69E-4
	GO:0017016	Ras GTPase binding	3.69E-4
	GO:0051020	GTPase binding	3.69E-4
	GO:0017048	Rho GTPase binding	3.69E-4
Cellular component	GO:0030054	cell junction	1.94E-5
	GO:0005911	cell-cell junction	9.19E-5
	GO:0043229	intracellular organelle	8.44E-4
	GO:0070161	anchoring junction	8.78E-4
	GO:0005886	plasma membrane	9.49E-4

Figure S7

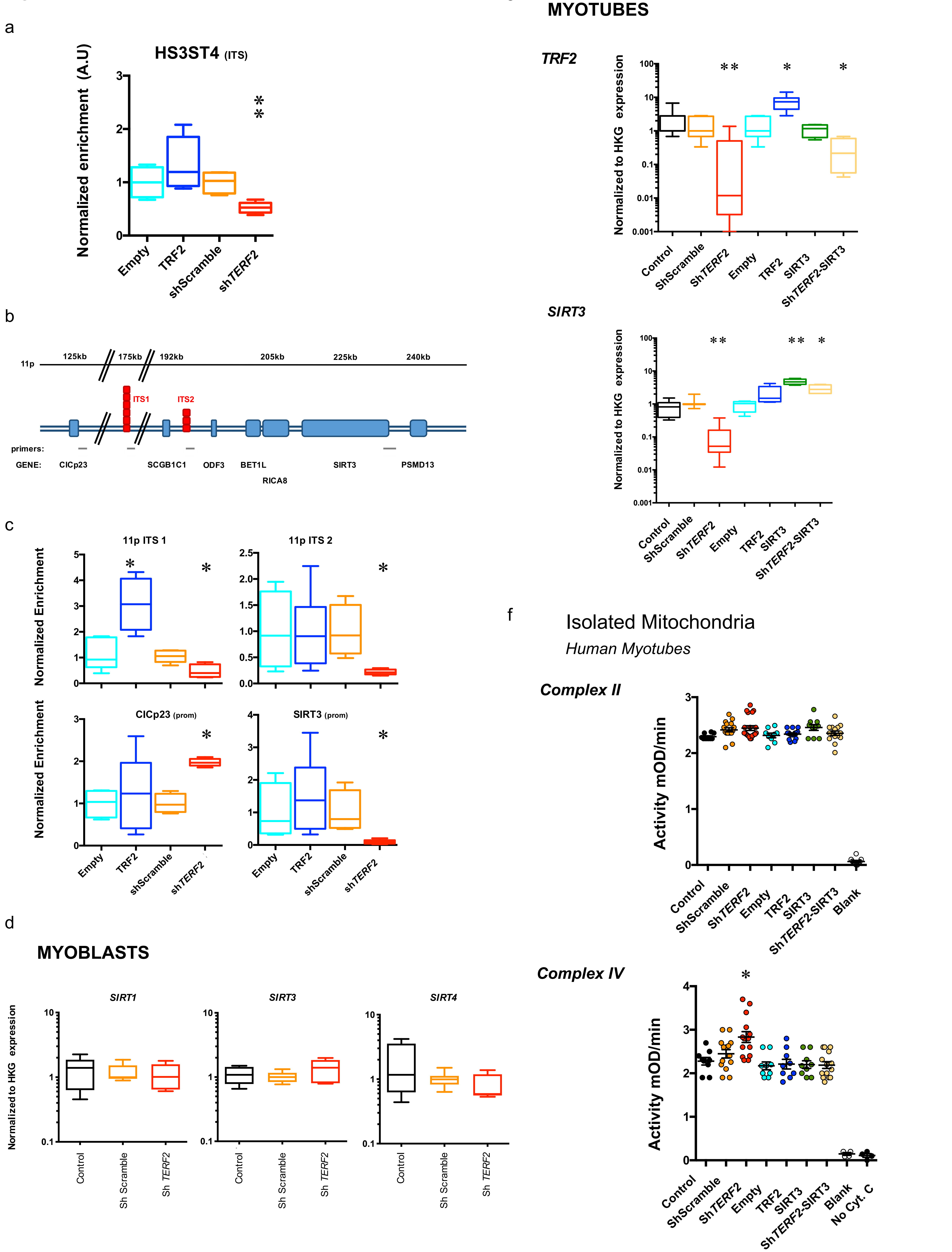

a
HS3ST4 (ITS)
Normalized enrichment (A.U)
Empty
TRF2
shScramble
shTERF2
b
11p
125kb
175kb
192kb
205kb
225kb
240kb
ITS1
ITS2
primers:
GENE:
ClCp23
SCGB1C1
ODF3
BET1L
RICA8
SIRT3
PSMD13
c
11p ITS 1
11p ITS 2
ClCp23 (prom)
SIRT3 (prom)
Normalized Enrichment
Empty
TRF2
shScramble
shTERF2
d
MYOBLASTS
SIRT1
SIRT3
SIRT4
Normalized to HKG expression
Control
Sh Scramble
Sh TERF2
e
MYOTUBES
TRF2
SIRT3
Normalized to HKG expression
Control
ShScramble
ShTERF2
Empty
TRF2
SIRT3
ShTERF2-SIRT3
f
Isolated Mitochondria
Human Myotubes
Complex II
Complex IV
Activity mOD/min
Control
ShScramble
ShTERF2
Empty
TRF2
SIRT3
ShTERF2-SIRT3
Blank
No Cyt. C

Figure S8

a

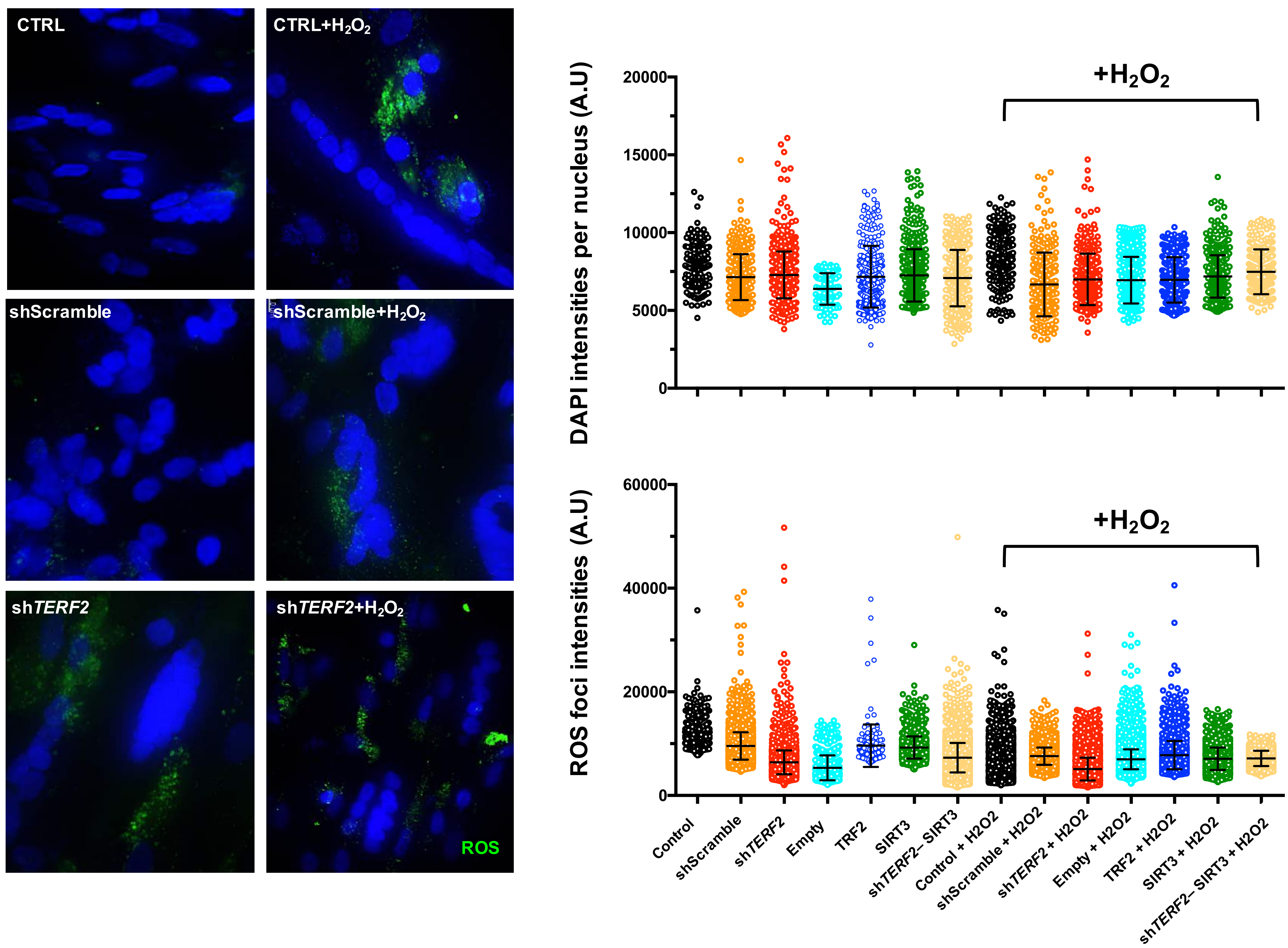
CTRL
CTRL+H_2O_2
shScramble
shScramble+H_2O_2
sh*TERF2*
sh*TERF2*+H_2O_2
ROS
DAPI intensities per nucleus (A.U)
20000
15000
10000
5000
0
+H_2O_2
ROS foci intensities (A.U)
60000
40000
20000
0
+H_2O_2
Control
shScramble
sh*TERF2*
Empty
TRF2
SIRT3
sh*TERF2*– SIRT3
Control + H2O2
shScramble + H2O2
sh*TERF2* + H2O2
Empty + H2O2
TRF2 + H2O2
SIRT3 + H2O2
sh*TERF2*– SIRT3 + H2O2

b

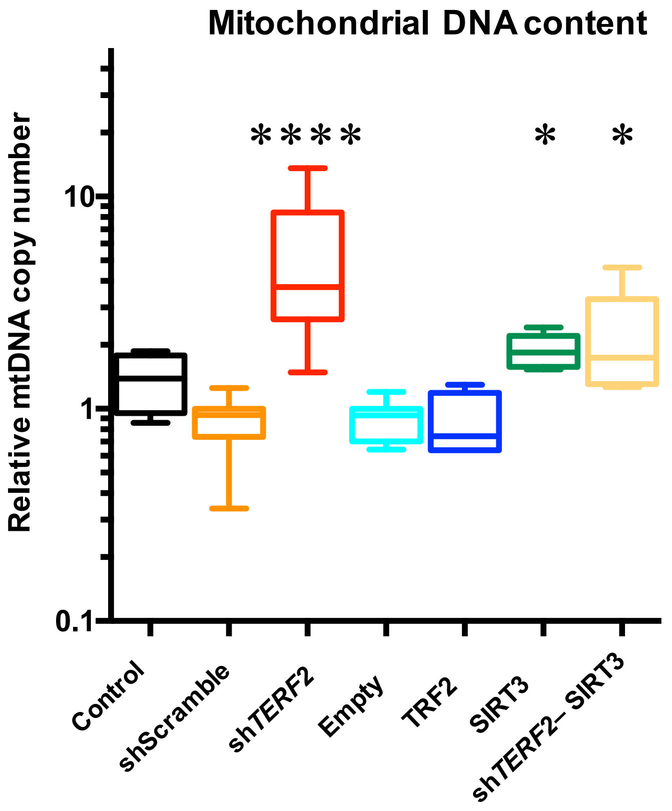
Mitochondrial DNA content
Relative mtDNA copy number
10
1
0.1
****
*
*
Control
shScramble
sh*TERF2*
Empty
TRF2
SIRT3
sh*TERF2*– SIRT3

Figure S9

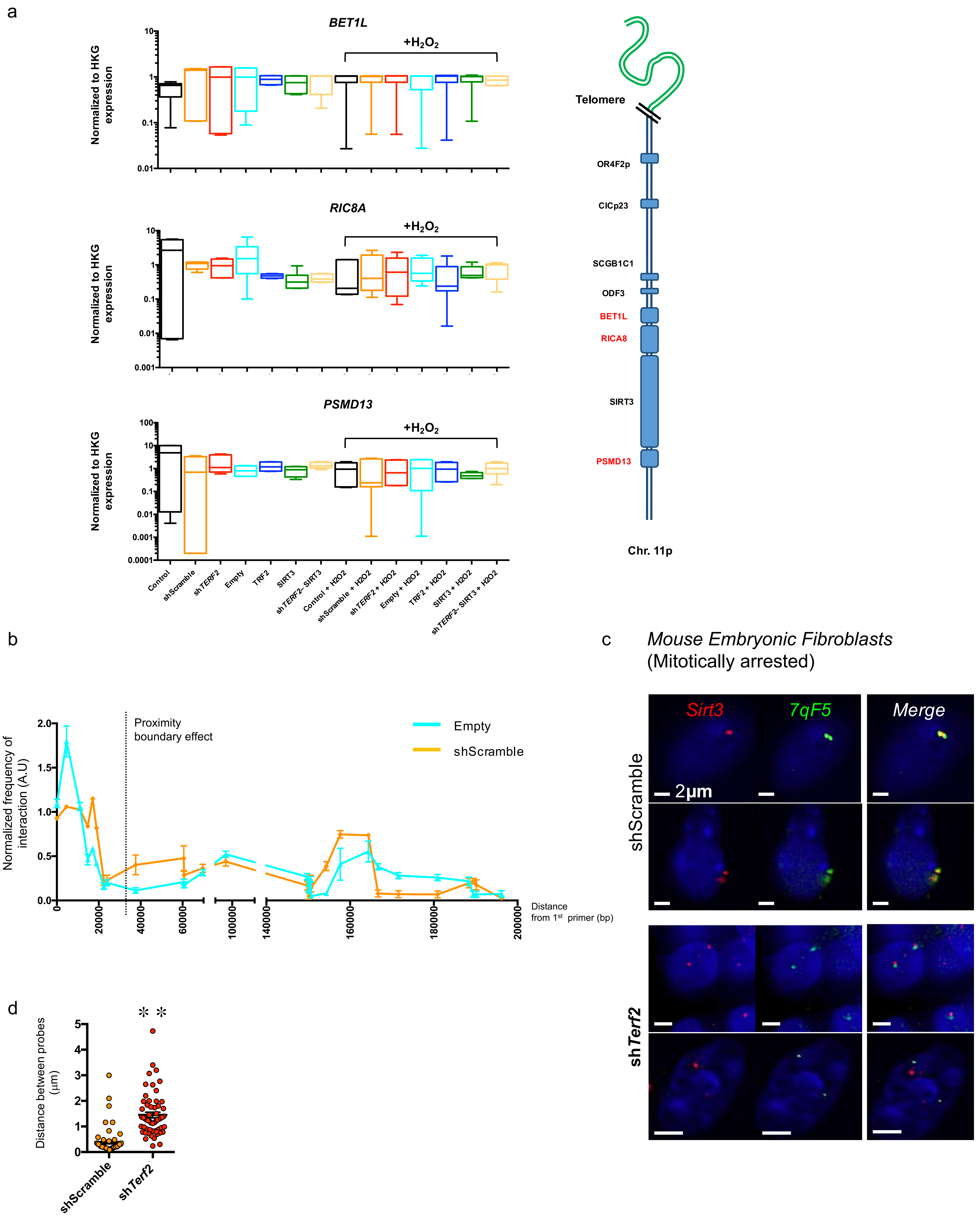
a
BET1L
+H2O2
Normalized to HKG expression
RIC8A
PSMD13
Control
shScramble
shTERF2
Empty
TRF2
SIRT3
shTERF2- SIRT3
Control + H2O2
shScramble + H2O2
shTERF2 + H2O2
Empty + H2O2
TRF2 + H2O2
SIRT3 + H2O2
shTERF2- SIRT3 + H2O2
Telomere
OR4F2p
CICp23
SCGB1C1
ODF3
BET1L
RICA8
SIRT3
PSMD13
Chr. 11p
b
Proximity boundary effect
Empty
shScramble
Normalized frequency of interaction (A.U)
Distance from 1st primer (bp)
c
Mouse Embryonic Fibroblasts
(Mitotically arrested)
Sirt3
7qF5
Merge
shScramble
2µm
shTerf2
d
Distance between probes (µm)
shScramble
shTerf2

Figure S10

a

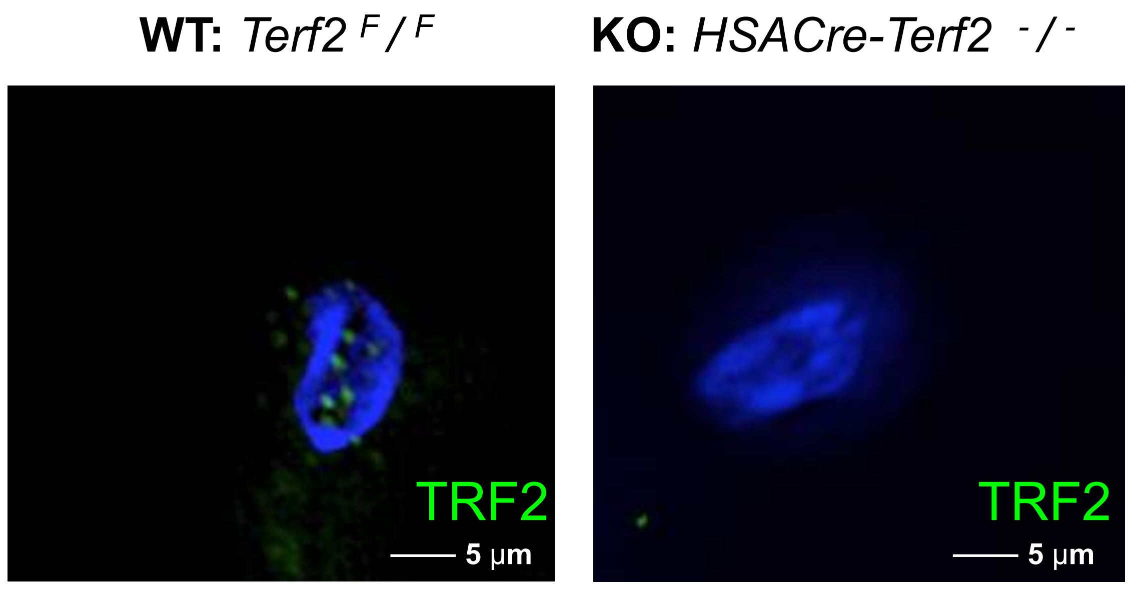

b

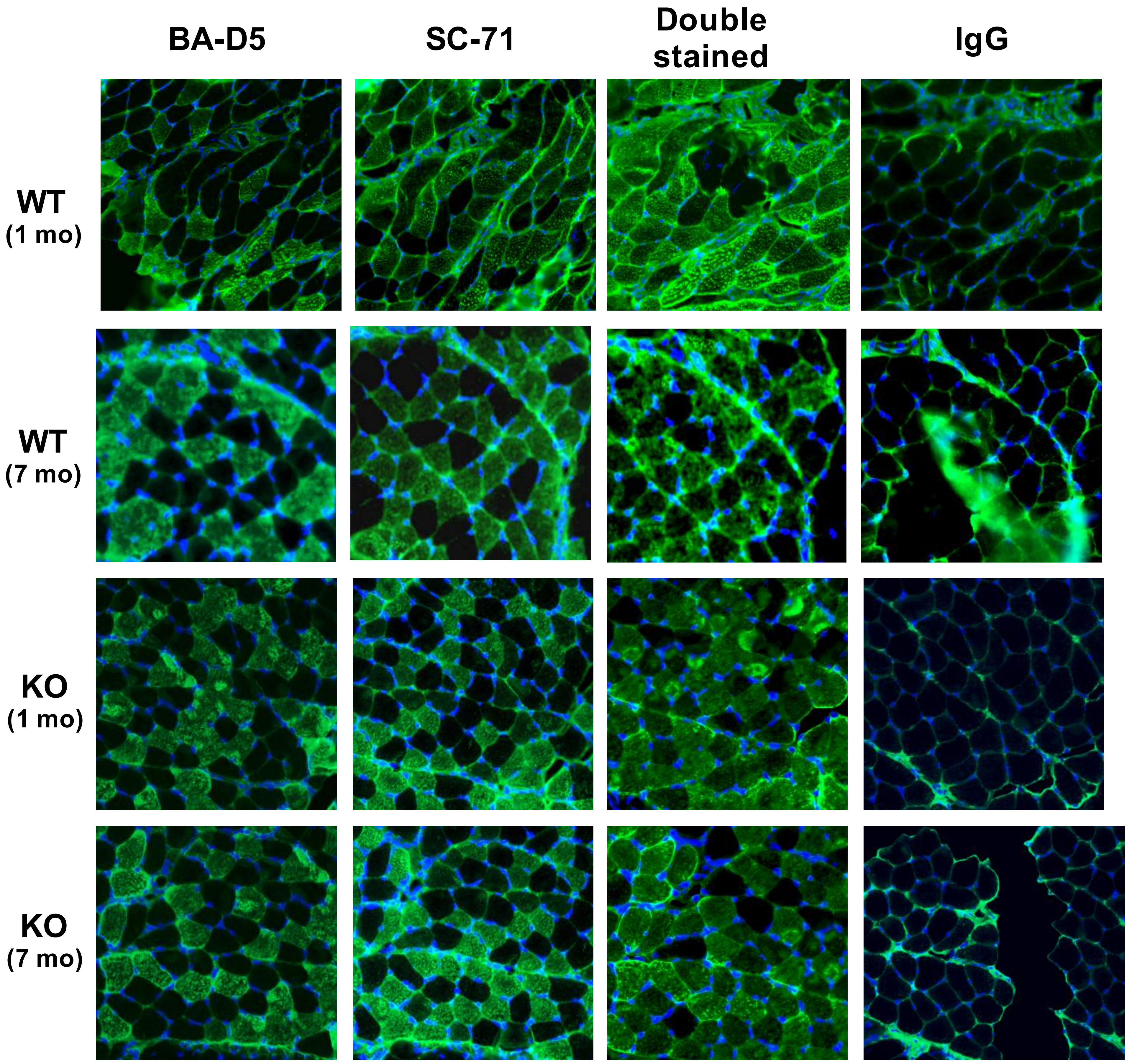

BA-D5 staining : fiber Type I determination

SC-71 staining : fiber Type IIa determination

Double staining : unidentified fiber Type (as not stained fibers)

IgG : negative control

Figure S11

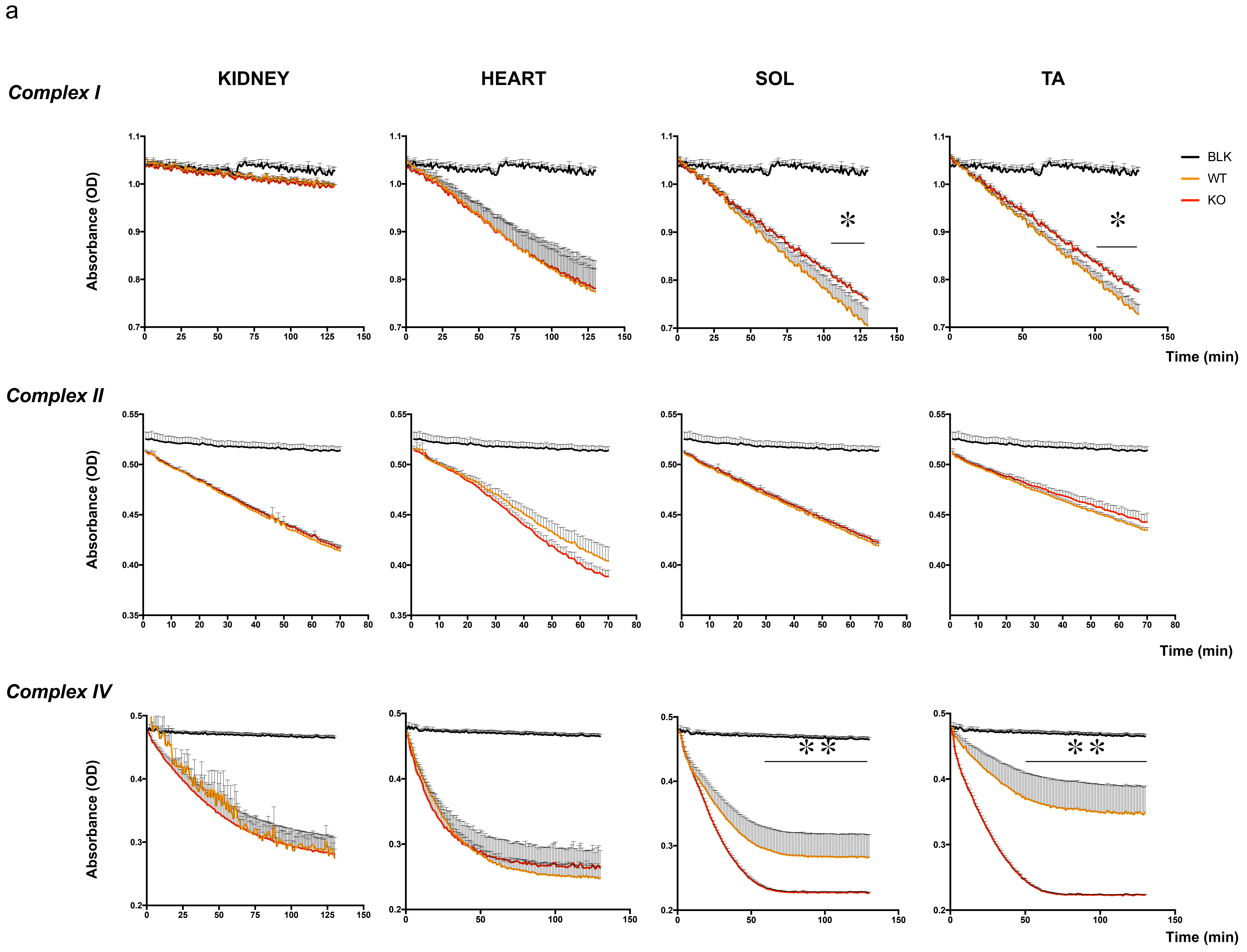
a
KIDNEY
HEART
SOL
TA
Complex I
Complex II
Complex IV
Absorbance (OD)
Time (min)
BLK
WT
KO
*
**

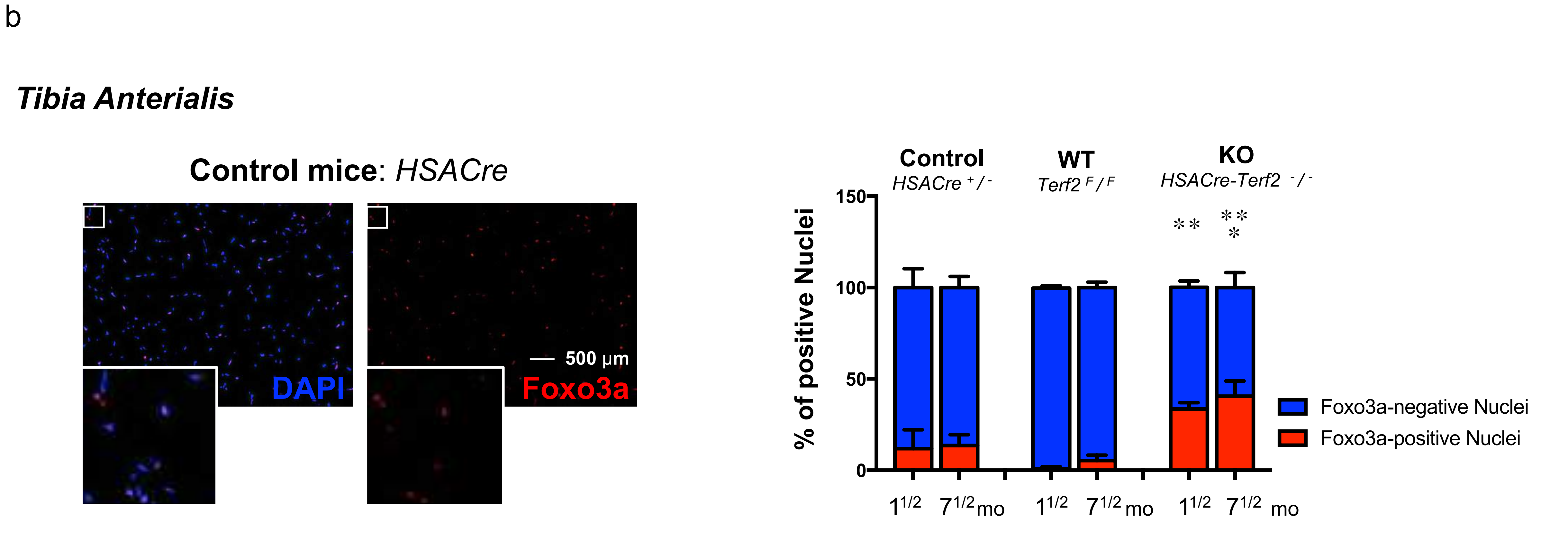
b
Tibia Anterialis
Control mice: HSACre
DAPI
Foxo3a
500 µm
Control
HSACre +/-
WT
Terf2 F/F
KO
HSACre-Terf2 -/-
% of positive Nuclei
Foxo3a-negative Nuclei
Foxo3a-positive Nuclei
1 1/2
7 1/2 mo
**
***
