# Supplementary material for: Mitochondrial function in skeletal myofibers is controlled by a TRF2‐SIRT3 axis over lifetime
Source: Aging Cell. 2020 Jan 28;19(3):e13097. doi: 10.1111/acel.13097 (PMC7059141; doi:10.1111/acel.13097)
Supplement: Supplementary file 3 [file ACEL-19-e13097-s003.docx]

**FULL METHODS**

**Human biopsies and Ethic statement**

The collection of fetal muscle biopsies was approved by the “Agence Française de la Biomedecine” of the Ministery of Health for legal access to the biological material in full accordance with the law (research protocol number PFS13-006). Samples were obtained after therapeutic abortion. Parents have provided written informed consent for the use of biopsies for medical research in accordance with the Declaration of Helsinki. Muscle biopsies were processed by fetopathologists from fetuses not affected by a muscular pathology. Skeletal muscle biopsies from teens and adults were obtained from the Nice Hospital (CHU l’Archet registered as protocol number DC-2015 2374) and from the Tumorothèque, Assistance Publique des Hôpitaux de Marseille, agreement n°AC-2013-1786, from voluntary healthy donors using standardized muscle biopsy protocol. All biopsies were tested for musculoskeletal pathologies with negative outcome.

**Mice –*Transgenic model*:** In order to generate a skeletal muscle specific TRF2 KO mice (*HSACre^+^ / ^-^* - *Terf2 ^-^ / ^-^*) two characterized stains were crossed. First, we obtained a TRF2 floxed mice from the Jackson laboratory (strain B6; 129P2-*Terf2*^tm1Tdl^/J) that have been previously characterized^1^. TRF2 floxed mice (noted *Terf2 ^F^ / ^F^*) were first backcrossed with C57BL/6 strain for 6 generations to homogenate the genetic background. The mice were then crossed with the *HSACre* ^+^/^-^ strain expressing the Cre-recombinase under the control of the Human Skeletal Actin (*HSA*) gene promoter specifically expressed in striated muscle fibers^4^. The study design and protocols were controlled and approved by the Comité Institutionnel d‘Éthique Pour l‘Animal de Laboratoire (CIEPAL) and the Ministry of Education and Scientific Research under the number APAFIS#10444-2017052414468355v2. All experiments were performed at the ANIPHY core (ENS, Lyon), agreement n°C693880803.

**Mitochondrial DNA quantification:** DNA was extracted using a high salt precipitation and resuspended in 50μl of TE (10mM Tris HCl pH8.0; 1mM EDTA). After DNA quantification, all samples were diluted to a concentration of 3ng/μl in TE. A qPCR assay was then performed using 1μl of the prepared diluted samples. All samples were run in triplicates and biological triplicate for human myotubes (n=9 per condition); 4 mice per group (n=12 per condition) using FastStart universal SYBR Green master Mix (Roche) and a 7900HT Fast Real-time PCR system with an 384 well block module (Applied Biosystem). Melting curves were analyzed to exclude nonspecific amplification products. The program used is as followed: denaturation 95ºC – 5min, followed by 40 cycles of 95°C – 30 sec, 60°C – 30s annealing, 72°C – 30s extension. PCR was stopped with a final 98°C – 10 min step. We used a set of primers specific to human as described elsewhere^2^, to measure the relative mitochondrial content (dubbed mtDNA and nucDNA, respectively; See Extended primer list). To determine the mitochondrial DNA content, we used the following equations: ∆CT = (nucDNA C_T_ – mtDNA C_T_); Relative mitochondrial DNA content = 2x2^∆CT^.

**Mitochondrial Complex Enzyme Activity Assay:** following manufacturers’ instructions (Abcam) mitochondrial extracts were prepared either from snap freeze tissues in isopentane/Liquid nitrogen double bath as commonly used for biochemistry assays (i.e., TA, SOL, GASTR, Heart and Kidney) or directly from freshly harvested transduced human myotubes and processed within 4 hours. Briefly, tissues were reduced into powder using a liquid nitrogen cooled mortar and pestle apparatus (Bel-Art Scienceware) and homogenize subsequently with a dounce homogenizer in ice cold PBS. After supernatant collection, a small portion was extracted and used to determine protein concentration. The remaining solution was then adjusted to a 5.5mg/ml final concentration in PBS. Proteins were then extracted from sample by adding 1:10 of detergent solution as supplied by the manufacturer. Solution was left on ice for 30min to allow solubilization of mitochondria and thereafter centrifuged for 20min at 12000g at 4°C. Finally supernatant was aliquoted, snap freeze and stored at -80°C for storage. Aliquots were then used only once after thawing. 3 mitochondrial complexes were assessed: Complex I (MitoTox complex I OXPHOS Activity, abcam); Complex II (Complex II Enzyme Activity Microplate Assay Kit, abcam) and Complex IV (Complex IV Rodent Enzyme Activity Microplate Assay). For each assay, appropriate concentrations were determined for each tissue. We used 70ug of mitochondrial tissue extract for skeletal muscle, 100ug for Kidney and 50ug for Heart samples. Last, plates were prepared according manufacturers protocol, using technical duplicates, 2 wells for positive controls and 4 wells for background measures.

Kinetics were followed by ODs measures using a SPECTROstar Nano apparatus (BMG labtech). For each assay, a total of 10 mice (5 WT; 5 KO) were tested in duplicates, and two control tissue used (e.g., heart, kidney). Each data point represents the average of 10 measures. For human myotubes, assays were done in duplicate using biological triplicate for a total of 6 measures per data point (minimum). Activity was then deduced and reported by calculating the slope of the curve as suggested by the manufacturer (△OD/ Time), using only the region where a linear activity was observed.

**Mitochondrial SIRT3 Activity Assay:** following manufacturers’ instructions (Abcam ab156067) mitochondrial extracts were prepared from freshly harvested transduced human myotubes and processed within 4 hours. Briefly, cells were pelleted and lysed in mitochondrial extraction buffer, a small portion was then used to determine protein concentration. The remaining solution was adjusted to a 5.5mg/ml final concentration in PBS. Proteins were then extracted from sample by adding 1:10 of detergent solution as supplied by the manufacturer. Solution was left on ice for 30min to allow solubilization of mitochondria and thereafter centrifuged for 20min at 12000g at 4°C. Finally, supernatant was aliquoted, snap freeze and stored at -80°C for storage. Aliquots were then used only once after thawing. For each plate run, we used dilution series (10-30μg) of our enriched mitochondrial extracts along with: a provided positive control (pure Recombinant SIRT3); a buffer only control, a no enzyme control and a no NAD control (no substrate) well.

Kinetics were followed by Fluorescent measures (detector at 450nm; 1min intervals) using a GloMax Discover Microplate reader (Promega). For each assay, myotubes were done in duplicate using biological triplicate for a total of 6 measures per data point (minimum). Activity was then deduced and reported by calculating the slope of the curve as suggested by the manufacturer (△Fluorescence/ Time), using only the region where a linear activity was observed.

**Western Blot:** Cells were scraped from culture dish in 1X PBS complemented with protease inhibitors, mixture was spin down (800g, 10min at 4°C) and pellet stored at -80°C for further use. Whole cell lysates were prepared from cells by adding cell lysis buffer (i.e., Muëller Buffer: 50mM Hepes pH7.4, 0.1% Triton X-100, 4mM EGTA pH8.0, 10mM EDTA pH8.0, 15mM Na_4_P_2_O_7_, 25mM NaF, 5mM NaVO_4_, 100mM **β**-glycerophosphate) complemented with phosphatase inhibitors (Roche). For human biopsies, samples were first reduced into powder using a liquid nitrogen cooled mortar and pestle apparatus (Bel-Art Scienceware) and homogenize subsequently using a Precellys®24 apparatus (Cayman chemical) with ceramic beads before being briefly sonicated. Total protein concentration was determined using a Bradford Protein Assay reagent (BioRad), by measuring absorption at 750nm on a spectrophotometer. For each sample, 30μg of protein was resolved in a 4-15% gradient mini-protean precast polyacrylamide gels (BioRad) and transferred to PVDF low fluorescence membranes (Millipore) for 5 hours at 4°C (Wet transfer). After blocking for 1 hour with 10% skim milk in PBST (0.2% Tween-20 in PBS), the membranes were incubated overnight at 4°C with primary antibodies diluted in 5% BSA in PBST. The following primary antibodies were used: TRF2, mouse monoclonal, (clone IMG-124A, 1:1000, Imgenex); TRF2, rabbit monoclonal, (clone EPR3517(2), 1:2000, Abcam); TRF1, mouse monoclonal (ab1423, 1:2000, Abcam); RAP1, rabbit monoclonal (A300-306A, 1:1000; Bethyl); POT1, rabbit monoclonal (NB500-176, 1:1000; NovusBiological); TIN2, rabbit monoclonal (ab136997, 1:1000; Abcam); TPP1, rabbit monoclonal (A303-069A, 1:1000; Bethyl); Actin, rabbit polyclonal, (1:3000, Santa Cruz); ATM, mouse monoclonal, (clone 2C1, 1:1500, Abcam); Ser 1981 phospho-ATM, mouse monoclonal, (clone 10H11.E12, 1:1500, Cell Signaling Technology); FoxO3a, rabbit monoclonal (clone 75D8, 1:1500, Cell Signaling Technology); HSP60, goat polyclonal (1:5000, Abcam); SIRT3, rabbit monoclonal (clone D22A3, 1:1500, Cell Signaling Technology); SIRT3, rabbit monoclonal (clone C73E3, 1:1500, Cell Signaling Technology); LC3A/B, rabbit polyclonal (1:2000, Cell Signaling Technology). The membranes were then rinsed three times in PBST for 10min and incubated 1 hour at room temperature with appropriate secondary antibodies diluted (1:15000) in Li-COR blocking reagent or 10% skim milk (e.g., IRDye^®^ 800CW/680 Goat anti-mouse; Goat anti-rabbit; Li-COR; HRP Goat anti-rabbit IgG; HRP Goat anti-mouse IgG; HRP Donkey anti-goat IgG; Vector Laboratories). Following three rinses in PBST for 15min, membranes were imaged by IR fluorescence with the Odyssey^®^ imaging system (Li-COR) or Fusion Solo imaging system (Vilber). For comparison between membranes, quantifications were normalized to the intensity of the ladder (2.5μl, PageRuler^Tm^ plus prestained protein ladder; ThermoScientific).

**Cell culture:** Cells used for this study were isolated^3^ from patient 12 (Extended Data. 1) and produced as previously described^4^. For day-to-day maintenance, human myoblasts were seeded in dishes coated with 0.1% pigskin gelatin in 4:1 Dulbecco modified Eagle medium/Medium 199 supplemented with 15% FBS, 0.02M HEPES, 1.4mg/l vitamin B12, 0.03mg/l ZnSO_4_, 0.055mg/l dexamethasone, 2.5μg/l hepatocyte growth factor and 10μg/l basic fibroblast growth factor. Cultures were maintained in a 5% oxygen environment and passaged at ~60% confluency. Population doublings (PDs) were calculated as PD = ln[(final number of cells)/(initial number of cells)]/In(2).

Myogenicity of the cells was verified by myotubes formation following a change to differentiation medium (2% horse serum in 4:1 Dulbecco modified Eagle medium: Medium 199) when 70-90% confluent.

For infection of myotubes (Figure 1D), cells (myoblasts) were seeded in 10 cm dishes, switch to differentiation media (2% Horse Serum) upon confluence (90%) and transduced at least week after. Cells were transduced at a MOI 2 using the different shRNAs and vectors. For ectopic upregulation, we used previously validated constructs and associated control (annoted TRF2 and Empty)^5^. For SIRT3, we used the same backbone and cloned the *SIRT3* cDNA sequence in lieu of *TERF2* cDNA. For downregulation experiments, we used MISSION shRNAs (pLKO.1; Sigma) with references as followed: *TERF2* (TRNC0000004809; TRNC0000004810; TRNC0000004812 and TRNC0000004813), *FOXO3A* (TRNC0000010335 and TRNC00000235491) and control (SHC002).

**RT-qPCR:** Cells were lysed (RNeasy plus kit (Qiagen)) after washing with PBS, scraped (BD Biosciences) and sheared by centrifugation through Qiashredder columns (Qiagen). Total RNA purified according to the manufacturer’s instructions was quantified on a Nanodrop 1000 spectrophotometer (Thermo Scientific). For Reverse Transcription (RT) 2x 500ng RNA was reverse transcribed in technical duplicates using two separate kits (SuperScriptIII, invitrogen; High Capacity cDNA RT Kit, Applied Biosystem). The cDNA was diluted 1:4 in water for quantitative RT-PCR (qRT-PCR) in triplicates using FastStart universal SYBR Green master Mix (Roche) and a 7900HT Fast Real-time PCR system with 384 well block module (Applied Biosystem). Melting curves were analyzed (SYBR green) to exclude non-specific amplification products. We confirmed amplicon size at least once on agarose gels. Crossing-threshold (Ct) values were normalized by subtracting the geometric mean of three housekeeping genes (GAPDH, PPIA and HPRT1). All Ct values were corrected by their PCR efficiency, determined by 1:2 or 1:4 cDNA dilution series.

**Immunofluorescence Assays:** Traditional immunofluorescence assays were performed as followed: cells were grown on cover slides and fixed for 10min on ice with 4% paraformaldehyde in PBS. After PBS washes, cells were incubated for 1h at room temperature in blocking solution (1%Triton X-100, 1%BSA, 5% donkey serum in PBS). Cells were then stained overnight at 4°C in blocking solution containing the respective primary antibodies (TRF2, 1:100; Foxo3A, 1:250; LaminB, 1:200). After three washes with PBS/ 0.1% Triton X-100, slides were incubated for 1h30min at room temperature with secondary antibodies (AlexaFluor 1:500) in PBS containing 0.5% Triton X-100, 5% BSA. Slides were mounted with Vectashield with DAPI (Vector Laboratories, Burlingame, USA). Images were taken using a Delta vision elite system (GE) with a 60X oil-immersed lens (60X/TRIF – Plan Apochromat; Olympus).

***Telomeric Induced Foci (TIFs)*:** Cells were grown on cover slides and fixed for 10min on ice with 4% paraformaldehyde in PBS. After PBS washes, cells were incubated for 1h at room temperature in blocking solution (1%Triton X-100, 1%BSA, 5% donkey serum in PBS). To perform the PNA-FISH staining, cells were washed twice with SSC2X for 5min at RT and subsequently treated with RNaseA for 45min at 37°C. After additional SSC2X washes (5min, 4°C), coverslides were dried and incubated upside-down with a hybridization solution containing the PNA probe (20μl H_2_O; 70μl formamide; 7μl 10% blocking B (Roche); 1μl 1M Tris pH7.2; 1μl probe) and sealed on coverslips using rubber cement. Slides were then heated at 85°C for 4min, and incubated in the dark at 37°C in a humidification chamber for 2 hours. After removal of the rubber cement, cells were serially washed in three different solutions: twice for 15min at RT with washing solution I (10mM Tris pH 7.2; 70% formamide); twice for 15min at RT with washing solution II (150mM NaCl; 50mM Tris pH7.2; 0.05% Tween 20) and twice with PBS for 5min at RT. Cells were then blocked for 1 hour with the blocking solution and immuno-stained overnight at 4°C in the blocking solution containing the primary rabbit polyclonal anti-53BP1 antibody (Novus Biologicals, 1:500). After three washes with PBS/ 0.1% Triton X-100, slides were incubated for 1h30min at room temperature with Alexa 555 Donkey anti-rabbit secondary antibody in PBS containing 0.5% Triton X-100, 1% BSA, 2.5% donkey serum. Slides were mounted with Vectashield with DAPI (Vector Laboratories, Burlingame, USA). Images were taken using a DeltaVision elite system (GE). Co-localization events, representing telomeric DNA damages (TIFs), were counted in at least 30 nuclei per condition from three independent experiments using the IMARIS software.

***Reactive Oxygen Species (ROS):*** Cells were grown on cover slides and treated as indicated in the ROS kit instructions (Enzo). Briefly, transduced differentiated cells were washed twice and 1ml of fresh differentiation media was added, with or without drugs and incubated for 30min at 37°C (N-Acetyl-L-cystein: 5mM; EGCG: 10mM; H_2_O_2_: 100μM). After additional wash and media renewal (1ml), cells were incubated for 1 hour at 37°C with a solution composed 2X ROS detection and 4μl of Oxidative stress reagent per 10ml (5mM; dilution 1:2500). The solution was added to the 1ml of fresh media to adjust to the appropriate concentration. Cells were then washed three times with 1X PBS and directly mounted using 15μl of vectashield+DAPI (no fixation). Pictures were taken using a DeltaVision Elite system (GE). An average of 100 stacks and 50 nuclei were taken per conditions. Images were then treated using IMARIS. Intensities of ROS foci and DAPI staining were used for analyses, excluding single-nuclei cells for myotubes analysis.

***Mitochondrial network:*** Cells were grown on cover slides and treated as indicated in the MitoTracker^®^ Red CMXRos manual. Briefly, transduced differentiated cells were washed twice and incubated with MitoTracker (25nM) diluted in 1ml of fresh differentiation media, with or without drugs and incubated for 30min at 37°C (EGCG: 10mM; H_2_O_2_: 100μM). After additional washes (3 x 5min in PBS), cells were counter stained with anti-LaminB antibody. Cells, were blocked for 1 hour with blocking solution and immuno-stained overnight at 4°C in a solution containing the primary rabbit polyclonal anti-LaminB antibody (Abcam, 1:500). Next, cells were washed with PBS/ 0.1% Triton X-100, slides were incubated for 1 hour at room temperature with Alexa 488 Donkey anti-rabbit secondary antibody in PBS containing 0.5% Triton X-100, 5% BSA. Slides were mounted with Vectashield containing DAPI (Vector Laboratories, Burlingame, USA). Images were taken using a DeltaVision elite system (GE).

***Fiber type characterization:*** Mice were sacrificed, fresh muscle were dissected (e.g., *gastrocnemius-soleus* and *tibia anterialis*) and then flash frozen (dry-ice and liquid nitrogen mixture) in an OCT block. Transverse frozen sections (10μm) of each muscle mice were prepared and keep at -80°C for storage. In order to characterize fiber types, we used two mouse antibodies directed against myosin MyHC-I (Isoform BA-D5, DSHB, University of Iowa) and MyHC-IIa (Isoform SC-71, DSHB, University of Iowa) specific to type I and type IIa fibers, respectively.

Staining procedure went as follow: muscle slides were melted at room temperature for 30min and fixed for 10min with 4% paraformaldehyde in PBS. After three PBS 1X 3 min washes, muscle slides were incubated for permeabilization in 0.5% Triton X-100 in PBS for one hour and washed again three time in PBS 1X. Slides were Blocked using a solution of M.O.M IgG diluent (Vector Laboratories, Burlingame, USA) for one hour at room temperature. Slides were then briefly washed twice in PBS 1X for 5 min and incubated with M.O.M protein diluent solution for 5min. Next, slides were incubated with primary antibodies in M.O.M protein diluent (1:20) over night at 4°C. Each slides were treated simultaneously with 4 conditions separated using a Dako pen (Agilent); MyHC-I, MyHCIIa, combined MyHC-I + MyHC-IIa and no primary; to allow characterization of fibers in 3 categories; I, IIa and Unidentified, respectively. After three washes of 5 min each in PBS 1X, slides were incubated for 40min in M.O.M protein diluent containing the secondary antibody (Alexa 488 Donkey anti-mouse; 1:200) and washed three times 5min in PBS 1X. Last, slides were mounted with Vectashield containing DAPI (Vector Laboratories, Burlingame, USA). Images were taken using an Axio Imager 2 and a 20X lens (Zeiss)

***3D DNA FISH:*** Three dimensional DNA Fluorescent in situ Hybridization was performed as previously described elsewhere ^6^. For human myotubes we used a PNA C-Rich probe along with a probe generated by Nick translation using the BAC clone RP11-656018 (CHORI) as template. For MEFs, BACs targeting the *SIRT3* and subtelomeric 7q locus were used as template: B6Ng01-185F08 and B6Ng01-137I17, respectively.

**ChIP and ChIP-Seq:** Samples for chromatin immunoprecitpitation (IP) were prepared as followed. IP using TRF2 antibody (TRF2- Imgenex124A) were crosslinked for 10 min at RT and 20 min at 4°C with 0.8% formaldehyde (methanol free, ultrapure EM grade, Polysciences, Inc; Warrington PA). Reaction was stop at RT for 10 min with the addition of Glycine to a final concentration of 0.125 M. Cells were rinsed twice with ice-cold 1X PBS, scraped from the dish and pelleted after centrifugation (800g, 5min at 4°C). Next, cells were treated according to the manufacturer’s guidance (Pierce Classic Protein G IP Kit, Thermo Scientific). For sonication, we used a total processing time of 15min per sample in a Bioruptor (Diagenode) using the following settings: 14 cycles; 30 Sec ON/30 Sec OFF on High power. Sonicated DNA was controlled on a 2% agarose gel, adequate sonication is achieved when a smear ranging from 200-700bp is obtained. IPs were processed using a 4°C O/N incubation (concentration of TRF2 antibody at 1.5μg); 1μl of each preparation: IP, IgG, Rabbit non-immune Serum, No crosslink control, no Antibody control and 1% input were used as controls for ddPCR analysis. Primers were designed for the promoter region of each gene, results are normalized to inputs and Alu repeats and normalized to adequate controls (e.g., Empty for TRF2, shScramble for sh*TERF2*). Each PCR primer pairs were tested on genomic DNA to verify specificity and efficiency (see primer list file).

**ChIP-Seq analysis:** DNA was sequenced on an Illumina HiSeq in single-end mode with a read length of 49nt, producing an average amount of 2.5 Million of reads per replicate and 3.8 Million for the 1% input replicates. Raw data were filtered and trimmed using Trimmomatric^7^ reducing the reads set to -3.8% per file. Reads from each file were aligned to the human reference genome hg38 using Bowtie2^8^ with default parameters. The aligned files from identical samples (replicates) were then merged together and all subsequent analysis were performed using MACS^9^ and a suits of tools including BEDTools^10^ and BEDOPS^11^. Significant peaks (p<0.05) were identified and annotated using the UCSC database (hg38). Data, including raw files and annotated peaks have been deposited on NCBI Gene Expression Omnibus (GEO; <http://www.ncbi.nlm.nih.gov/geo/)>, accession GSE88983 and the list of genes associated peaks is reported in Supplemental Items 1.

**Chromatin Conformation Capture 3C**: Cell cross-linking and lysis: 4 x 10cm dishes were plated with cells and grown in muscle media until 60% confluence (average of 25 million cells). Cells were then washed once at 37°C in Solution A prior to fixation with a fresh mixture of muscle media with 0.8% formaldehyde (methanol free, ultrapure EM grade, Polysciences, Inc; Warrington PA) at RT on a shaking plateform for 10min. The reaction was stopped with 1.25M Glycine (Sigma # G88980) with agitation 10min at RT and 15min on ice. Supernatant was discarded and dishes were washed twice with 10ml ice-cold Solution A. 550μl of cell lysis buffer (10mM Tris-HCl pH 8.0, 10mM NaCl, 0.2% Ige CaI CA 630 (NP-40) + 50μl protease inhibitor Sigma #P8340) was added to each dish on ice. Cells were scraped and collected in a 35ml falcon tube and spun at 300g for 10min, 4°C. Supernatant was discarded and fresh 1.1ml of 3C Lysis Buffer was added. Cells were resuspended, transferred into a 1.5ml tube and incubated on ice for 20min (chemical lysis), then mechanically lysed using a Dounce homogenizer (two 10 up and down strokes separated by 1min incubation on ice). Finally cells were spun 5min at 300g, supernatant discarded and cell pellet washed twice with 300μl of NEB2 buffer (50mM NaCl, 10mM Tris-HCl pH 7.9, 10mM MgCl2, 1mM DTT). The pellets were stored at -80°C.

Chromatin digestion: Cells suspended in a 300μl of NEB2 buffer were split in 3x 100μl tubes and an additional 262μl NEB2 buffer 1X was added to each tube. To remove un-cross-linked proteins, 38μl of 1% SDS was added to the tubes and incubated at 65°C for 10min in a thermo-shaker. SDS was quenched by addition of 44μl 10% Triton X-100 on ice (final volume of 444μl per tube). 5μl aliquots were taken per samples as undigested controls. Chromatin was then digested overnight at 37°C in a thermo-shaker by adding 400 Units of HindIII (NEB) per samples. The next day, tubes were put on ice, and 5μl aliquot per samples were taken and labeled ‘digestion control’. To determine the digestion efficiency, control aliquots (digested and un-digested) were de-crosslinked by adding 10μl proteinase K (NEB) in 90μl 10mM Tris-HCl pH 7.5 and incubated at 65°C for 2 hours. The digestion efficiency was then analyzed on 0.6% agarose gels. Successful digestion is visualized as a smear with the majority of the fragments between 5 to 10Kb.

Ligation: Ligation mixes were made in 15ml falcon tubes. Each ligation tube contained an 8ml mixture composed of 800μl 10% Triton X-100, 800μl 10x ligation buffer (500mM Tris-HCl pH 7.5, 100mM MgCl2, 100mM DTT), 80μl BSA (10mg/ml NEB), 80μl ATP (100mM, Invitrogen) completed with ddH_2_O. Digested chromatin was transferred to each appropriate tube and 1U of T4 DNA ligase was added to each tube (cohesive ligation) and left overnight at 16°C.

DNA extraction: 25μl of 20mg/ml proteinase K was added to each tube and incubated at 65°C during the day. Next, an additional 25μl was added, and the reaction continued overnight. The next day, the 8ml reaction was cooled to RT and transferred into 50ml VWR ultra high performance tubes. 800μl of 3M Na Acetate pH 5.5 (Ambion) and 18ml of 100% ethanol was added to each tube. DNA was precipitated 2 hours at -20°C. DNA was pelleted by spinning the tubes for 1 hour at 4°C at 12,000g. Pellets were washed ten times with 70% ethanol, and finally resuspended in 500μl TE (10mM Tris pH 8.0, 0.1mM EDTA). RNA was removed by adding 1μl of 1mg/ml RNaseA for 2 hours at 37°C. DNA was purified a second time by phenol:chloroform extraction. Briefly, 500μl phenol (pH 8) was added to the 500μl sample and vortexed for 30s. Samples were spun down in a tabletop centrifuge at max speed for 5 min. The upper layer was then transferred to a new tube and mixed with a fresh 500μl of phenol: chloroform mixture (1:1). Tubes were vortexed another 30s, spun down and supernatant transferred to a new tube where 500μl chloroform was added. Tubes were vortexed for 30s, spun down and the supernatant was transferred to a new 1.5ml tube. The phenol:chloroform procedure was repeated twice. 1/10 volume of 3M Na acetate was added to the 400-500μl supernatant retrieved (40 to 50μl) and completed with 1ml of 100% ethanol. Tubes were inverted several times to properly mix the content and left at 4°C overnight. DNA was pelleted by spinning down the tubes at max speed for 30 min at 4°C. DNA pellets were washed with 70% ethanol 3 times to insure salt removal (in case salts were not removed, an agarose dialysis step was added to the procedure). DNA was re-suspended in a final volume of 40μl TE.

Ligation efficiency: 3C ligation efficiency was checked by running an aliquot of each tube on 0.8% agarose gels.

**3C quantification:** 3C samples were prepared as described above. Quantification of interactions was done using the ddPCR technology (Biorad) following the manufacturers’ instructions with the following modifications in the PCR profile set-up: denaturation 95ºC – 10min, followed by 40 cycles of 94°C – 30 sec, 60°C – 30s annealing, 72°C – 30s extension. PCR was stopped with a final 98°C – 10 min step. Plates were then read on the ddPCR droplet reader. All primers were HPLC purified (list available on demand), and a tail probe strategy was used to detect amplicons by adding a short DNA sequence to the SubTel primer^12^, when an internal probe was not available. All PCR pairs used were tested and amplicons were topo-cloned in order to validate the sequences amplified. To quantify and normalized samples, we used the 8 first primer pairs located within the 20Kb limit (Proximity effect) of the fixed primer. An average of the 8 was made and used to normalize 3C target interactions.

**TRF analysis:** Terminal restriction fragment assay was done as previously described^4^.

**Statistical Analysis:** All experiments were repeated at least three times, with three biological replicates (with the exception of human biopsies). Quantitative data are displayed as means ± standard error of the mean. Sample sizes as well as the statistical test used of each experiment are described in each corresponding figure legends or methods. Results from each group were treated with the GraphPad prism software for all statistical tests. All tests were two-sided and alpha set at 0.05. Only p-values less than 0.05 were considered statistically significant.

NOTE – References are specific to supplemental methods.

1. Celli, G. B. & de Lange, T. DNA processing is not required for ATM-mediated telomere damage response after TRF2 deletion. *Nature Cell Biology* **7,** 712–718 (2005).

2. Rooney, J. P. *et al.* PCR based determination of mitochondrial DNA copy number in multiple species. *Methods Mol. Biol.* **1241,** 23–38 (2015).

3. Robin, J. D. *et al.* Isolation and immortalization of patient-derived cell lines from muscle biopsy for disease modeling. *J Vis Exp* 52307 (2015). doi:10.3791/52307

4. Robin, J. D. *et al.* Telomere position effect: regulation of gene expression with progressive telomere shortening over long distances. *Genes Dev.* **28,** 2464–2476 (2014).

5. Biroccio, A. *et al.* TRF2 inhibits a cell-extrinsic pathway through which natural killer cells eliminate cancer cells. *Nature Cell Biology* **15,** 818–828 (2013).

6. Robin, J. D. *et al.* SORBS2 transcription is activated by telomere position effect-over long distance upon telomere shortening in muscle cells from patients with facioscapulohumeral dystrophy. *Genome Res.* **25,** 1781–1790 (2015).

7. Bolger, A. M., Lohse, M. & Usadel, B. Trimmomatic: a flexible trimmer for Illumina sequence data. *Bioinformatics* **30,** 2114–2120 (2014).

8. Langmead, B. & Salzberg, S. L. Fast gapped-read alignment with Bowtie 2. *Nat. Methods* **9,** 357–359 (2012).

9. Zhang, Y. *et al.* Model-based analysis of ChIP-Seq (MACS). *Genome Biol.* **9,** R137 (2008).

10. Quinlan, A. R. & Hall, I. M. BEDTools: a flexible suite of utilities for comparing genomic features. *Bioinformatics* **26,** 841–842 (2010).

11. Neph, S. *et al.* BEDOPS: high-performance genomic feature operations. *Bioinformatics* **28,** 1919–1920 (2012).

12. Robin, J. D., Ludlow, A. T., LaRanger, R., Wright, W. E. & Shay, J. W. Comparison of DNA Quantification Methods for Next Generation Sequencing. *Sci Rep* **6,** 24067 (2016).
