# Supplementary material for: Mitochondrial function in skeletal myofibers is controlled by a TRF2‐SIRT3 axis over lifetime
Source: Aging Cell. 2020 Jan 28;19(3):e13097. doi: 10.1111/acel.13097 (PMC7059141; doi:10.1111/acel.13097)
Supplement: Supplementary file 5 [file ACEL-19-e13097-s005.docx]

**Legend to the Supplementals**

**Figure S1.**

**a.** Recapitulative table of biopsies used in the study. Patient’s sex, age at biopsy and localization of punction are reported. All patients were healthy and free from muscle-related diseases. **b.** Slot blot and associated quantifications of relative telomere signal from human skeletal biopsies normalized to Alu repeats. Telomere length decreases in human skeletal biopsies with age (Fetuses *vs.* Adults (17/35), p=0.27; Adults *vs.* Elders (60/72), p=0.046; Fetuses *vs.* Elders, p=0.002; Kruskal-Wallis multiple comparison test; $\alpha$=0.05). **c.** TRF2 protein level correlates with age in skeletal muscle biopsies. The 95% intervals of confidence are indicated by discontinuous blue lines as well as the equation and associated goodness of fit (R^2^). **d.** Immunoblots and associated quantifications (bottom) of whole protein extracts from human biopsies collected at different ages with antibodies against TRF2, TRF1, TPP1, TIN2, RAP1, POT1 and B-ACTIN used as loading control (lower panel). Biopsies were grouped in 3 categories: Fetuses; Young Adults (17-35 yo) and Elders (>60 yo).

**Figure S2.**

**a.** Gene expression quantified by RT-qPCR in transduced myoblasts and myotubes**.** Cells were collected 5 and 10 days after transduction for myoblasts and myotubes respectively. Each measure represents the average fold-change expression of six independent repetitions (technical RT duplicates of biological triplicates) normalized to three housekeeping genes (HKG: *HPRT*, *PPIA* and *GAPDH*); △△Ct method). Means $\pm$ SEM with associated statistical significance are reported for myoblasts and myotubes (Mann-Whitney multiple comparison test and Kruskal-Wallis multiple comparison test, respectively; $\alpha$=0.05). **b.** Fusion index characterization of myotubes transduced and/or treated by N-acetyl-L-Cysteine (NAC), Epigallocatechin gallate (EGCG), two antioxidant or hydrogen peroxide (H_2_O_2_). Fusion index was calculated as the average number of nuclei counted per myotube at day 17 of differentiation in 10 independent and randomly chosen microscope fields (DeltaVision; GE). Single nucleus cells represented <5% of cells in all conditions. Means ± SEM are shown. **c.** Immunoblots and associated quantifications of whole protein extracts from human myotubes transduced with sh*TERF2* and associated control (shScramble), collected at day25 with antibodies against TRF2, TRF1, TPP1, TIN2, RAP1, POT1 and alpha-Tubulin used as loading control.

**Figure S3.**

**a.** 53BP1 staining and TIFs analysis in transduced myotubes using four different ShRNAs against *TERF2.* No statistical difference was seen between the different conditions (ANOVA, Kruskal-Wallis multiple comparison test; $\alpha$=0.05). Single nucleus cells were excluded from the analysis (*e.g.,* less than 5% of total values). **b.** Immunoblots and associated quantifications of transduced human myotubes (shScramble and *shTERF2*; respectively) using alpha-Tubulin as loading control. We report the protein levels of DNA Damage Response (DDR) associated proteins (*i.e.,* p53, ATM and phosphorylated counterparts). No difference was observed between conditions, suggesting that TRF2 depletion in myotubes does not trigger the DDR pathway. **c.** Telomere length was measured by Telomere Restriction Fragment analysis (TRF). TRF from myotubes transduced with the different constructs used in the study. No differences in telomere length were observed upon transduction.

**Figure S4.**

**a.** ROS-related foci intensities in transduced myotubes using the ROS-iD® detection kit (GE). An average of 100 z-stacks were taken for each condition (Deltavision Elite®, GE). Pictures from 10 to 15 independent and randomly chosen microscope fields were taken, then treated post-acquisition and deconvoluted with IMARIS. Single nucleus-cells were excluded from the analysis. DAPI intensities (top) and ROS related foci intensities (middle) are reported. All intensities fall into the same range. Outliers represented less than < 5% of the total intensities as shown by overlapping Means ± SEM. Normalized quantification of ROS in transduced myotubes (bottom) shown as total number of ROS foci normalized to the number of nuclei (from 10-15 independent microscope fields). Hydrogen peroxide treatment increases ROS production as reported by the increased ROS foci number (Empty vs. Empty+H202, p= 0.0274 ; Empty *vs.* TRF2+H202, p= 0.0291 ; Holm-Sidak's multiple comparisons test; 𝛼=0.05). Down regulation of TRF2 significantly increases ROS (shScramble *vs.* sh*TERF2*, p< 0,0001; Holm-Sidak's multiple comparisons test; 𝛼=0.05) and antioxidant treatments (*e.g.,* NAC, EGCG) reduces ROS to control condition’s levels. **b.** IF staining of FOXO3A (red), in transduced myotubes using 4 shRNAs against *TERF2*. Associated quantification of positive FOXO3A nuclei observed per conditions is reported. Single nucleus cells were excluded from the analysis (*e.g.,* less than 5% of total values). Means ± SEM are shown. **c.** FOXO3A representative western blots, staining and associated quantifications in transduced myotubes. Relative protein level quantifications normalized to B-ACTN and control (Empty and shScramble as reference for TRF2 overexpression and sh*TERF2* conditions; respectively; A.U, arbitrary units). All assays were run in biological triplicates. Means $\pm$ SEM are shown. In post-mitotic cells, increase or decrease in TRF2 level induces a stress response with FOXO3A modulation (shScramble vs. sh*TERF2*, p=0.0098; Empty vs. TRF2, p= 0.03; Man-Whitney test; $\alpha$=0.05). **d.** Kinetics of mitochondrial complex I, II and IV activity in transduced myotubes as reported by the apparatus before activity calculations. Sh*TERF2* transduced myotubes exhibit specific mitochondrial defects. We report a decreased complex I activity (p<0.001) and an increased complex IV activity (p<0.001, Kolmogorov–Smirnov’s test; α=0.05). Activity was deduced and reported in Figure 2 by calculating the slope of the curve as suggested by the manufacturer (△OD/ Time), using only the time period where a linear activity was observed.

**Figure S5.**

**a.** Mitochondrial network in transduced and chemically-treated myotubes was analyzed using Mitotracker^®^. An average of 100 z-stacks were taken for each condition (Deltavision Elite®, GE). Pictures from 10 to 15 independent and randomly chosen microscope fields were taken, treated post-acquisition and deconvoluted with IMARIS. Single nucleus cells were excluded from the analysis. Nuclei were stained using an Anti-Lamin B antibody (Green) and counterstained with DAPI. A punctate mitochondrial staining (observed as fragmented orange dots) is observed in myotubes transduced with sh*TERF2* suggesting mitochondrial fission. Similarly, H_2_O_2_ treatments induce a punctuated mitochondrial staining (*i.e.,* a discontinuous orange staining, lacking global homogeneity along the myotubes structures). **b.** NAD+/NADH enzymatic staining of transduced myotubes. Nicotinamide adenine dinucleotide-nitro-blue tetrazolium reductase (NADH-TR) staining performed in transduced myotubes. Dark coloration corresponds to the deposition of purple formozan precipitate at sites of mitochondrial activity. Typically, mitochondria-rich tissues (*i.e.,* Oxidative fibers, Type I fibers) will display a darker staining than those relying on glycolysis (*i.e.,* Glycolitic fibers, Type II). TRF2 depleted myotubes exhibit a positive strong coloration, corresponding to higher oxidation of NADH, thus advocating for an oxidative phenotype (as classically seen for Type I fibers).

**Figure S6.**

**a.** Percentage of reads containing TTAGGG repeat motifs. Motifs were searched in raw fastq files, without considering mismatches and including reverse complement motifs. All files from the Illumina sequencing were evaluted (i.e., 3 biological replicates for each of the 4 conditions). Corresponding SRA accessions identifiers are displayed on top of the bars. The % of telomeric reads varies in parallel with TRF2 expression modulation, confirming the TRF2 specificity of the ChIP-seq experiments. **b.** Peak distributions. Annotations are provided from RefSeq database and assigned using Homer software. Graph 1 shows the peaks founded in shTERF2 samples replicates versus shScrable Peaks; graph2 peaks from surexpressed TRF2 experiment vs Empty samples. Peaks are represented in 3 groups: (i) in blue those founded in both conditions (i.e., same overlapping positions) labeled as “conserved peaks”; (ii) in red the peaks founded in only one condition (shTERF2 and TRF2) labeled as gained peaks; in yellow the peaks only presented in the control condition (ShScramble and Empty) labeled as “lost peaks”. **c.** Top canonical pathways generated with IPA (IPA Q Ingenuity®Systems, Redwood City, USA). The IPA program analyzes input list of genes provided against the Ingenuity Pathway Knowledge Base (IPKB), derived from known functions and interactions of genes published in the literature, allowing one to identify global biological networks, functions and pathways altered from a given dataset. We used genes-lists encompassing genes located within <5000 nt of identified peak(s). List 1 represents the top canonical pathways given by IPA using the genes/peaks loss upon TERF2 downregulation (shTERF2 vs. shScramble); list 2 the top canonical pathways using the genes/peaks gained in TRF2 overexpression (TRF2 vs. Empty). We have also use genes related to theses 2 lists of top pathways to generated the over represented GO terms from Gene Ontologie (GO) database, using GOrilla software (http://cbl-gorilla.cs.technion.ac.il/). GO terms, descriptions and corresponding P-values are represented in 2 tables. Interestingly, in both gain and loss conditions, the top pathways are related to GPCR/cAMP/PKA/CREB signaling, suggesting a role of TRF2 in modulating this pathway in skeletal muscle cells.

**Figure S7.**

**a**. Validation of the TRF2 Chip-Seq. We used primers encompassing the *HS3ST4* TRF2 binding site (shScramble *vs.* sh*TERF2*, p= 0.0095; Mann-Whitney comparison test, $\alpha$=0.05). **b.** Schematic representation of the localization of the Internal Telomeric Sequences (ITS) at the 11p locus with surrounding genes and primers used for ChIP-ddPCR^®^ reported in **c.** Quantification of TRF2 enrichment by ChIP-ddPCR^®^ at the subtelomeric *SIRT3* region. Results were normalized to Alu repeats. At distal sites, TRF2 enrichment is decreased upon *TERF2* knock-down (shScramble *vs.* sh*TERF2*: 11pITS1, p=0.041; SIRT3 (prom), p= 0.0386; Mann-Whitney comparison test, $\alpha$=0.05), whereas the most subtelomeric site (*CICp23*) is enriched (shScramble *vs.* sh*TERF2*, p= 0.032). TRF2 enrichment is increased upon *TERF2* overexpression (Empty *vs.* TRF2, p= 0.024). **d.** Gene expression quantified by RT-qPCR in transduced myoblasts (as presented in Figure 1) normalized to three housekeeping genes (HKG: *HPRT*, *PPIA* and *GAPDH*; △△Ct method). N=6 per condition (technical duplicates of biological triplicates), means $\pm$ SEM with associated statistical significance are reported (Kruskal-Wallis multiple comparison test; $\alpha$=0.05). *TERF2* depletion reduces transcription of *SIRT6* without modulating other main Sirtuins and correlates with increased DNA damage as reported by TIFs assay (Figure 1). **e.** Gene expression quantified by RT-qPCR in transduced myotubes normalized to housekeeping genes (HKG: *HPRT*, PPIA and *GAPDH*; △△Ct method). N=6 per condition (technical duplicates of biological triplicates), means ± SEM with associated statistical significance are reported (Kruskal-Wallis multiple comparison test; α=0.05). **f.** *SIRT3* rescue experiments using enriched mitochondria extracts from transduced human myotubes. Mitochondrial complex II and IV activity. *SIRT3* overexpression in TRF2-depleted myotubes restores the mitochondrial-associated activity (Complex IV: shScramble *vs.* sh*TERF2*-SIRT3, p >0.9; Holm-Sidak's multiple comparisons test; $\alpha$=0.05). ✻ p< 0.05**;** ✻✻ p< 0.01.

**Figure S8.**

**a.** ROS-related foci intensities in transduced myotubes using the ROS-iD® detection kit (GE). An average of 100 z-stacks were taken for each condition (Deltavision Elite®, GE). Pictures from 10 to 15 independent and randomly chosen microscope fields were taken, treated post-acquisition and deconvoluted with IMARIS. Single nucleus cells were excluded from the analysis. DAPI intensities (top) and ROS related foci intensities (bottom) are reported. All intensities fall into the same range. Outliers represented less than < 5% of the total intensities as shown by overlapping Means ± SEM. **b.** Relative quantification of mitochondrial DNA content in transduced myotubes. Mitochondrial DNA (mtDNA) was quantified by qPCR and normalized to genomic DNA from three independent experiments (△△Ct method; tRNA-Leu and B2-microglobulin for mt and nuclear DNA; respectively). Sh*TERF2* and *SIRT3* transduced myotubes show an increase in mitochondrial DNA content (shScramble *vs.* sh*TERF2*, p<0.0001; shScramble *vs.* *SIRT3*, p=0.03; shScramble vs. sh*TERF2*-SIRT3, p=0.025; Kruskal-Wallis multiple comparisons test; 𝛼=0.05). Importantly, the results suggest a partial rescue of *TERF2* downregulation by *SIRT3* overexpression (ROS foci, average mitochondrial DNA content). Means ± SEM with associated statistical significance are reported. ✻ p< 0.05**;** ✻✻✻✻ p< 0.001.

**Figure S9.**

**a.** Gene expression of genes located at the 11p locus as represented (left), in transduced myotubes. Genes surrounding *SIRT3* (in red) were quantified by RT-qPCR. Each measure represents the average fold-change expression of six independent repetitions (technical RT duplicates of biological triplicates) normalized to three housekeeping genes (HKG: *HPRT*, *PPIA* and *GAPDH*; △△Ct method). No statistical difference was found amongst conditions (Kruskal-Wallis multiple comparison test; α=0.05). **b.** Chromatin Conformation Capture (3C) performed on the 11p locus (first 2Mb) in myotubes as reported in Figure 4. Cells were collected 10 days after transduction. Each measure represents the amplification of interactions involving a fixed primer and a second primer along the 2Mb of the locus, both located in proximity of a *Hind*III restriction site. No statistical difference was detected between control conditions (Empty *vs*. shScramble; Unpaired t Test; $\alpha$=0.05). **c.** 3D-DNA FISH in transduced mouse embryonic fibroblasts (MEFs) mitotically arrested using mitomycin and associated quantification **d.** MEFs were transduced with a sh*Terf2* construct and corresponding control vector then labeled with a *Sirt3* (red) and a unique distal sub-telomeric 7q (green) probe (5Mb apart); N>40 nuclei, means ± SEM are shown. As in human myotubes, we observe a significant increase in separated signals (corresponding to an increased distance between the 7q telomere and the mouse *Sirt3* locus) in MEFs transduced with sh*Terf2* (Two-tailed Student’s t-test; α=0.05).

**Figure S10.**

**a.** *Terf2* immunostaining in transversal section of a WT and KO mice. *Terf2* staining was detectable in all of the WT mice nuclei but only in a portion of the KO, an expected result as the Cre activation is restricted to mature muscle fibers. **b.** representative fiber type characterization in *HSACre ^+^/ ^-^ -Terf2 ^-^ / ^-^* mice using myosin heavy chain antibodies against type I (BA-D5) and IIa (SC-71) fibers. We report here the serial staining (Type I, Type IIa, Type I+ Type IIa and IgG control, from left to right; respectively) performed to discriminate the fiber type composition of the *soleus* (SOL) in 4 different mice. Undetermined fibers (reported U Figure 5h) represent fibers that remained unstained after a double stained procedure.

**Figure S11.**

**a.** Mitochondrial complex II, I and IV activity in 40 weeks-old transgenic WT and KO; freshly harvested tissues (e.g., Heart; Kidney; *gastrocnemius*; *soleus* and *tibia anterialis*). 5 mice from each group were processed in duplicates using the Enzyme Activity Microplate Assay Kit (abcam) and kinetics recorded by a SPECTROstar Nano™ apparatus (BMGlabtech). No statistical differences were observed between WT and KO mice in mitochondrial complex II activity as well as activity of complex I and IV from heart and kidney tissues. **b.** *Foxo3a* staining and associated quantification in the TA by immunofluorescence. KO mice exhibit a higher percentage of positive Foxo3a nuclei, no statistical differences were seen between the *HSACre* and WT (*Terf2 ^F^ / ^F^* ) mice (Kruskal-Wallis multiple comparisons test; 𝛼=0.05)*.* ✻ <0,05; ✻✻ < 0,001; ✻✻✻ < 0,0001
